# Thirteen simple lifestyle scores and risk of cancer, cardiovascular disease, diabetes, and mortality: Prospective cohort study in the UK Biobank

**DOI:** 10.1002/ijc.70064

**Published:** 2025-07-24

**Authors:** Jie Ding, Ben Schöttker, Hermann Brenner, Michael Hoffmeister

**Affiliations:** ^1^ Division of Clinical Epidemiology and Aging Research German Cancer Research Center (DKFZ) Heidelberg Germany; ^2^ Medical Faculty Heidelberg Heidelberg University Heidelberg Germany; ^3^ German Cancer Consortium (DKTK) German Cancer Research Center (DKFZ) Heidelberg Germany

**Keywords:** cancer, cardiovascular diseases, lifestyle, non‐communicable diseases, type 2 diabetes

## Abstract

Numerous simple lifestyle scores have been developed for specific non‐communicable diseases (NCDs). This research aimed to investigate and compare the associations of various lifestyle scores with the incidence and mortality of NCDs. In 76,399 participants from the UK Biobank, we investigated the associations of 13 lifestyle scores with the incidence and mortality of cancer, cardiovascular disease (CVD), type 2 diabetes (T2D), and a composite of these NCDs. Cox proportional‐hazards regression models were used to estimate hazard ratios (HRs) for associations between lifestyle scores and NCD outcomes. During a median follow‐up time of 10.5 years, 12,214 incident NCD cases and 2250 NCD deaths were documented. Higher lifestyle scores were generally associated with a reduced risk of overall NCDs (HRs ranging from 0.65 to 0.89) and NCD mortality (0.51–0.92). Cancer (HRs ranging from 0.72 to 0.98) and CVD (0.55–0.87) risk were less dependent on lifestyle behaviors than T2D (0.18–0.74). Notably, the top three scores associated with cancer outcomes included smoking as a component, and those for T2D included body mass index (BMI). For overall NCD outcomes, lifestyle scores including both smoking and BMI showed the strongest associations. Healthy Lifestyle Score and the Chronic Disease Risk Index were the overall best‐performing scores to predict NCD risk and mortality. These findings suggest that the use of lifestyle scores designed for a single disease group can be extended for predicting multiple NCDs and mortality. Both smoking and BMI should be included in lifestyle scores aiming to predict overall NCD risk and mortality for future research and recommendations.

## What's New?

Cancer, cardiovascular disease, and type 2 diabetes share common lifestyle‐related risk factors. This large‐scale prospective cohort study examined and compared the associations between 13 simple lifestyle scores and these major non‐communicable diseases. The results suggest that the use of lifestyle scores designed for a single disease group can be extended for predicting the incidence and mortality of multiple non‐communicable diseases. The study highlights the value of collaborative efforts to develop shared lifestyle recommendations and a common lifestyle score to reduce the burden of non‐communicable diseases. The inclusion of both smoking and body mass index as lifestyle factors is essential.

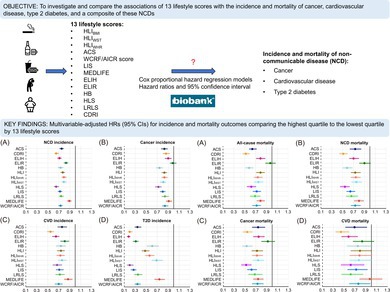

**Abbreviations**
ACS: American Cancer Society Guidelines Score
BMI: body mass index
CDRI: chronic disease risk index
CI: confidence intervals
CVD: cardiovascular disease
ELIH: empirical lifestyle pattern score for hyperinsulinemia
ELIR: empirical lifestyle pattern score for insulin resistance
HB: health behaviors score
HF: heart failure
HLI: healthy lifestyle index
HLI_WHR_
: healthy lifestyle index with waist‐to‐hip ratio
HLI_WST_
: healthy lifestyle index with waist circumference
HLS: healthy lifestyle score
HR: hazard ratios
LIS: lifestyle inflammation score
LRLS: low‐risk lifestyle score
MEDLIFE: Mediterranean lifestyle
MI: myocardial infarction
NCDs: non‐communicable diseases
SD: standard deviation
T2D: type 2 diabetes
VIF: variance inflation factor
WCRF/AICR: World Cancer Research Fund and the American Institute for Cancer Research score

## 1 INTRODUCTION

Non‐communicable diseases (NCDs), also known as chronic diseases, are the leading causes of death worldwide. NCDs accounted for 74% of all deaths (42 million) globally in 2019, with over 70% attributed to the major types of NCDs, including cancer, cardiovascular disease (CVD), and type 2 diabetes (T2D).^1^ At least 70% of premature CVD, T2D, and up to 40% of cancer cases were estimated to be caused by lifestyle‐related risk factors,^2^, ^3^, ^4^, ^5^ which highlights the pivotal role of promoting multiple lifestyle habits in NCD prevention.

Various recommendations proposed by organizations and evidence from prior studies guide individuals in adopting healthy lifestyle practices for NCD prevention. To measure adherence to these recommended habits, corresponding composite lifestyle scores have been developed as valid tools to evaluate the overall impact of lifestyles on health outcomes. Studies have repeatedly reported a strong inverse relationship between the number of healthy behaviors and the risk of incident events for which these scores were developed.^6^, ^7^ However, the variety of lifestyle guidelines and scores with different components and calculation methods has led to confusion. Understanding their relative predictive performance could inform better risk‐stratification tools and public health strategies. Furthermore, although some lifestyle scores were initially developed to assess associations with single disease entities, it is widely recognized that cancer, CVD, and T2D share common lifestyle‐related risk factors.^8^, ^9^, ^10^

Accordingly, we hypothesized that lifestyle scores originally developed to predict a single disease may also be eligible for risk assessment of other major NCDs. The objective of this study was to compare and benchmark 13 previously established lifestyle scores in terms of their associations with incident cancer, CVD, T2D, and a composite of these NCDs in the UK Biobank. We further sought to identify the common and distinct features of the top‐performing scores.

## 2 METHODS

### 2.1 Study population

The UK Biobank is a prospective cohort comprising over 500,000 participants aged between 40 and 69, recruited in 22 assessment centers across England, Wales, and Scotland from 2006 to 2010. Baseline data were collected through a self‐administered touch‐screen questionnaire, interviews, and physical measurements. Details about the study population have been extensively reported previously.^11^, ^12^

Of the 502,366 UK Biobank participants enrolled at baseline, participants were excluded without at least two 24‐h recall dietary assessments with plausible energy intake. Additionally, we excluded participants who withdrew from follow‐up, who were pregnant, not free of the assessed NCDs before the last 24‐h dietary recall assessment, lost to follow‐up before the last 24‐h dietary assessment, and without necessary data to create the lifestyle scores (Supplementary Figure 1). The final analysis included 76,399 participants.

### 2.2 Lifestyle scores

We included 13 lifestyle scores in the analysis, based on a previous systematic review that summarized lifestyle scores which have been used to investigate associations with various NCDs,^13^ including the American Cancer Society Guidelines Score (ACS), chronic disease risk index (CDRI), empirical lifestyle pattern score for hyperinsulinemia (ELIH), empirical lifestyle pattern score for insulin resistance (ELIR), health behaviors score (HB), healthy lifestyle index (HLI), healthy lifestyle index with waist‐to‐hip ratio (HLI_WHR_), healthy lifestyle index with waist circumference (HLI_WST_), lifestyle inflammation score (LIS), low‐risk lifestyle score (LRLS), Mediterranean lifestyle (MEDLIFE), World Cancer Research Fund and the American Institute for Cancer Research score (WCRF/AICR). Additionally, we included the healthy lifestyle score (HLS) that we previously developed for colorectal cancer.^14^

Detailed components and scoring criteria of the 13 lifestyle scores, along with references for each of the scores, are presented in Table 1 and Supplementary Table 1. HLI, HLS, and LRLS contained five lifestyle factors: smoking, alcohol consumption, diet, physical activity, and body mass index (BMI). ACS, CDRI, ELIH, ELIR, HB, LIS, and WCRF/AICR comprised four of these lifestyle factors. HLI_WST_ and HLI_WHR_ replace BMI in HLI with waist circumference and waist‐to‐hip ratio. MEDLIFE includes alcohol consumption, diet, physical activity, as well as nap, hours of sleep, watching TV, friends/family visits, and group activity. Details about dietary assessment are provided in the Supplementary Methods.

**TABLE 1:** Components of the 13 lifestyle scores.

Lifestyle score	Smoking	Alcohol intake	Diet	Physical activity	BMI	Other components	No. of components
ACS	N/A	X	X	X	X	N/A	4
CDRI	X	X	X	N/A	X	N/A	4
ELIH	N/A	X	X	X	X	N/A	4
ELIR	N/A	X	X	X	X	N/A	4
HB	X	X	X	X	N/A	N/A	4
HLI	X	X	X	X	X	N/A	5
HLI_WHR_	X	X	X	X	N/A	WHR	5
HLI_WST_	X	X	X	X	N/A	WST	5
HLS	X	X	X	X	X	N/A	5
LIS	X	X	N/A	X	X	N/A	4
LRLS	X	X	X	X	X	N/A	5
MEDLIFE	N/A	X	X	X	N/A	Nap, hours of sleep, watching TV, friends/family visits, group activity	8
WCRF/AICR	N/A	X	X	X	X	WST	5

Abbreviations: ACS, the American Cancer Society guidelines score; BMI, body mass index; CDRI, chronic disease risk index; ELIH, empirical lifestyle pattern score for hyperinsulinemia; ELIR, empirical lifestyle pattern score for insulin resistance; HB, Health behaviors; HLI, healthy lifestyle index; HLS, healthy lifestyle score; LIS, lifestyle inflammation score; LRLS, low‐risk lifestyle score; MEDLIFE, the Mediterranean lifestyle; N/A, not applicable; WCRF/AICR score, World Cancer Research Fund and the American Institute for Cancer Research score; WST, waist circumference; WHR, waist‐to‐height ratio.

### 2.3 Ascertainment of outcomes

The primary outcome included incidence and mortality of NCDs and their components. Incident NCDs included all types of cancer (excluding non‐melanoma skin cancer), major CVD (stroke, myocardial infarction (MI), and heart failure (HF)), and T2D, while NCD mortality was defined as death from cancer or total CVD. Secondary outcomes were specified for cancer and CVD. For cancer, these included incidence and mortality of lifestyle‐related cancer and other types of cancer. Lifestyle‐related cancers are defined as those that have shown significant associations with lifestyle scores in a previous systematic review,^13^ including breast, kidney, endometrial, ovarian, lung, esophageal, gastric, small intestinal, colorectal, anus and anal canal, pancreatic, gallbladder, and liver cancer. For CVD, secondary outcomes included individual incidence of stroke, MI, and HF. Given that the number of deaths from stroke, MI, and HF was too few to ensure sufficient statistical power, no analyses were performed for these outcomes. Detailed definitions and outcome codes are presented in Supplementary Methods and Supplementary Table 2.

Follow‐up for events was censored on the earliest date of the occurrence of study outcomes, the date lost to follow‐up, the date of death, or the end of follow‐up, whichever occurred first. Participant survival time was estimated from the last 24‐h dietary assessments until the censoring date. At the time of our analysis, hospital admission data were available up until October 31, 2022 for England, August 31, 2022 for Scotland, and May 31, 2022 for Wales. Death data were available up until November 30, 2022. Primary care data were available up until May 31, 2016 for England, March 31, 2017 for Scotland, and August 31, 2017 for Wales.

### 2.4 Statistical analysis

For all lifestyle scores, participants were divided into four categories based on the magnitude of their scores to ensure that the number of participants in each category was as equal as possible. The highest category represents the highest scores.

Baseline characteristics for each lifestyle score category were summarized as means (with standard deviation, SD) for continuous variables and numbers (percentages) for categorical variables. To assess and compare the performance of each lifestyle score, we evaluated the strength of their associations with the risk of developing or dying from primary and secondary outcomes using hazard ratios (HRs) and 95% confidence intervals (CIs) derived from Cox proportional hazard regression models. Scores were considered more predictive if they showed stronger inverse associations (i.e., lower HRs) with outcomes. Schoenfeld residuals were used to test the proportional hazards assumption, and the assumption was satisfied in each NCD subtype model. We created an additional level for the missing values of covariates.^15^ In the fully adjusted model, we adjusted for age at recruitment (years), sex (male or female), ethnicity (white, others, unknown), region (London, North West England, North‐Eastern England, Yorkshire and the Humber, West Midlands, East Midlands, South‐East England, South‐West England, Wales and Scotland), index of multiple deprivation (quintiles, missing data), household income (<£18,000, £18,000–£30,999, £31,000–£51,999, £52,000–£100,000, >£100,000, unknown), education (high degree (college/university degree or vocational qualification), middle degree (national examination at 17–18 years of age or national examination at 16 years of age), other qualifications, unknown), drug use (cholesterol‐lowering medication, blood pressure medication, and aspirin) (yes, no, unknown), and history of cancer screening (yes, no, unknown). For NCDs, the fully adjusted model was additionally adjusted for family history of diabetes, cancer, or CVD (yes, no, unknown). For disease‐specific outcomes, the fully adjusted model also included the family history of the respective disease. We calculated the variance inflating factor to assess the potential inflation of the variance of regression coefficients due to multicollinearity. All variance inflation factor (VIF) values of covariates were below 5 in the models. Subgroup analyses were conducted by age, sex, and index of multiple deprivation for NCDs. Potential interactions were assessed using the likelihood ratio test.

To facilitate comparability across the 13 lifestyle scores, the first category of the healthy lifestyle scores was used as the reference group with the least healthy lifestyle (ACS, HLI_BMI_, HLI_WHR_, HLI_WST_, HLS, LRLS, MEDLIFE, and WCRF/AICR). For lifestyle scores by defining the healthiest group as the reference (ELIH, ELIR, LIS, CDRI, and HB), we inverted the lifestyle scores so that HRs were comparable. Tests for linear trend were performed by incorporating the categories of each lifestyle score as a continuous variable into the multivariable Cox models.

All statistical tests were two‐sided, and statistical significance was defined as *p* <.05 or 95% CIs excluding 1.0. All the analyses were performed using SAS version 9.4 (SAS Institute Inc., Cary, NC, USA). The figures were plotted by R Statistical Software, version 4.2.1, with the package ggplot2.

## 3 RESULTS

### 3.1 Population characteristics

Of the 76,399 individuals included in this study, during a median follow‐up of 10.5 years, 12,214 participants developed one of the NCDs. Among them, 8334 participants developed cancer (4148 with lifestyle‐related cancer, 5620 with other cancer), 3535 participants developed CVD (1574 with MI, 1123 with stroke, and 1250 with HF), and 1698 participants developed T2D. There were 3056 deaths overall, of which 2250 were from the assessed NCDs. Among the included participants, 1687 died from cancer (1044 attributable to lifestyle‐related cancer, 643 to other cancers), 561 died from total CVD, and 2 died from T2D.

The baseline characteristics of the participants in the highest and lowest categories of the 13 lifestyle scores are provided in Table 2, and those for the full study population are described in Supplementary Table 3. The mean age at baseline was 55.5 years (SD = 7.8), and the majority of participants were white (*N* = 74,010, 96.9%). Compared to participants in the lowest category of the 13 lifestyle scores, those in the highest category were more likely to be women, non‐white, higher educated, and from areas with lower deprivation.

**TABLE 2:** Baseline characteristics of participants in the lowest and the highest categories of the 13 lifestyle scores.

Lifestyle score	Category	ACS	CDRI	ELIH	ELIR	HB	HLI	HLI_WHR_	HLI_WST_	HLS	LIS	LRLS	MEDLIFE	WCRF/AICR
N	C1	20,291	10,691	19,100	19,100	6241	18,742	17,951	17,751	16,379	23,527	15,852	21,839	17,995
	C4	13,528	12,335	19,199	19,199	18,341	18,753	21,609	17,649	8939	17,215	9429	23,741	16,822
Age (year), mean (SD)	C1	55.2 (7.8)	54.9 (7.9)	55.7 (7.7)	55.4 (7.8)	54.6 (7.7)	56.0 (7.5)	56.6 (7.5)	56.3 (7.5)	55.3 (7.7)	55.8 (7.7)	56.5 (7.4)	55.1 (7.8)	54.5 (7.8)
	C4	55.6 (7.9)	55.6 (7.9)	55.1 (7.9)	55.7 (7.8)	56.2 (7.8)	54.8 (8.2)	54.4 (8.1)	54.6 (8.1)	55.6 (8.0)	54.9 (8.0)	54.3 (8.1)	55.8 (7.9)	56.2 (7.7)
Female	C1	8026 (43.5)	4797 (44.9)	8007 (41.9)	7896 (41.3)	2068 (33.1)	8047 (42.9)	4648 (25.9)	6488 (36.6)	7313 (44.7)	10,478 (44.5)	9363 (59.1)	8686 (39.8)	6837 (38.0)
	C4	12,366 (64.1)	2289 (18.6)	13,145 (68.8)	12,126 (63.5)	12,235 (66.7)	11,736 (62.6)	16,517 (76.4)	12,201 (69.1)	5157 (57.7)	10,229 (59.4)	2617 (27.8)	15,294 (64.4)	11,319 (67.3)
White	C1	19,742 (97.3)	10,220 (95.6)	18,418 (96.4)	18,439 (96.5)	6098 (97.7)	18,350 (97.9)	17,590 (98.0)	17,408 (98.1)	15,940 (97.3)	23,019 (97.8)	15,411 (97.2)	21,138 (96.8)	17,559 (97.6)
	C4	12,950 (95.7)	12,078 (97.9)	18,600 (97.4)	18,635 (97.6)	17,658 (96.3)	17,945 (95.7)	20,689 (95.7)	16,848 (95.5)	8689 (97.2)	16,538 (96.1)	9195 (97.5)	23,020 (97.0)	16,107 (95.8)
Higher degree education	C1	133,326 (65.7)	6521 (61.0)	12,299 (64.4)	12,318 (64.5)	4000 (64.1)	12,343 (65.9)	11,877 (66.2)	11,737 (66.1)	10,153 (62.0)	15,162 (64.5)	10,129 (63.9)	13,894 (63.6)	11,601 (64.5)
	C4	8827 (65.3)	8688 (70.4)	12,790 (67.0)	12,674 (66.4)	12,448 (67.9)	12,237 (65.3)	13,938 (64.5)	11,365 (64.4)	6148 (68.8)	11,598 (67.4)	6447 (68.4)	16,138 (68.0)	11,351 (67.5)
Index of multiple deprivation Q1	C1	3843 (18.9)	1536 (14.4)	3206 (16.8)	3346 (17.5)	1106 (17.7)	3419 (18.2)	3379 (18.8)	3328 (18.8)	2680 (16.4)	4130 (17.6)	2698 (17.1)	4053 (18.6)	33,124 (18.4)
	C4	2752 (20.3)	2848 (23.1)	4094 (21.4)	3873 (20.3)	3650 (19.9)	3782 (20.2)	4217 (19.5)	3409 (19.3)	2066 (23.1)	3755 (21.8)	2141 (22.7)	4732 (19.9)	3344 (19.9)
Index of multiple deprivation Q3	C1	3942 (19.4)	1866 (17.4)	3693 (19.3)	3740 (19.7)	1136 (18.2)	3557 (19.0)	3379 (18.8)	3352 (18.9)	3084 (18.8)	4608 (19.6)	3016 (19.0)	4257 (19.5)	3463 (19.2)
	C4	2569 (19.0)	2426 (19.7)	3686 (19.3)	3699 (19.4)	3601 (19.6)	3566 (19.1)	4192 (19.4)	3437 (19.5)	1667 (18.7)	3217 (18.7)	1833 (19.4)	4581 (19.3)	3209 (19.1)
Index of multiple deprivation Q5	C1	4188 (21.2)	3084 (28.9)	4633 (24.3)	4413 (23.2)	1438 (23.0)	4055 (21.6)	3775 (21.0)	3755 (21.2)	3973 (24.3)	5130 (21.8)	3554 (22.5)	4591 (21.0)	3929 (21.8)
	C4	2489 (18.4)	1828 (14.8)	3342 (17.5)	3417 (17.9)	3332 (18.2)	3425 (18.3)	4052 (18.8)	3355 (19.0)	1383 (15.5)	2913 (17.0)	1450 (15.5)	4447 (18.7)	3183 (18.9)
Family history of cancer	C1	7089 (34.9)	3691 (34.5)	6713 (35.2)	6527 (34.2)	2180 (34.9)	6810 (36.3)	6480 (36.1)	6412 (36.1)	5862 (35.8)	8418 (35.8)	5745 (36.2)	7467 (34.2)	6164 (34.3)
	C4	4530 (33.5)	4201 (34.1)	6478 (33.9)	6636 (34.7)	6361 (34.7)	6170 (32.9)	7197 (33.3)	5813 (32.9)	2917 (32.6)	5587 (32.5)	3060 (32.5)	8150 (34.3)	5740 (34.1)
Family history of CVD	C1	11,373 (56.1)	5895 (55.1)	10,797 (56.5)	10,831 (56.7)	3344 (53.4)	10,721 (57.2)	10,214 (56.9)	10,102 (56.9)	9074 (55.4)	13,289 (56.5)	9186 (58.0)	11,732 (53.7)	9811 (54.5)
	C4	7409 (54.8)	6662 (54.0)	10,561 (55.3)	10,475 (54.8)	10,555 (57.6)	10,002 (53.4)	11,692 (54.1)	9521 (53.9)	4984 (55.8)	9351 (54.3)	5026 (53.3)	13,561 (57.1)	9655 (57.4)
Family history of T2D	C1	4133 (20.4)	2334 (21.8)	4351 (22.8)	4089 (21.4)	1108 (17.8)	3760 (20.1)	3433 (19.1)	3394 (19.1)	3307 (20.2)	4839 (20.6)	3298 (20.8)	4230 (19.4)	3540 (19.7)
	C4	2524 (18.7)	2024 (16.4)	3179 (16.7)	3206 (16.8)	3543 (19.3)	3417 (18.2)	4154 (19.2)	3396 (19.1)	1488 (16.7)	2974 (17.3)	1552 (16.5)	4551 (19.2)	3093 (18.4)

Abbreviations: ACS, the American Cancer Society guidelines score; CVD, cardiovascular disease; CDRI, chronic disease risk index; ELIH, empirical lifestyle pattern score for hyperinsulinemia; ELIR, empirical lifestyle pattern score for insulin resistance; HB, health behaviors; HLI, healthy lifestyle index; HLS, healthy lifestyle score; LIS, lifestyle inflammation score; LRLS, low‐risk lifestyle score; MEDLIFE, the Mediterranean lifestyle; WCRF/AICR score, World Cancer Research Fund and the American Institute for Cancer Research score; SD, standard deviation; T2D, type 2 diabetes.

### 3.2 Associations of the 13 lifestyle scores with NCD‐related incidence

**FIGURE 1:**
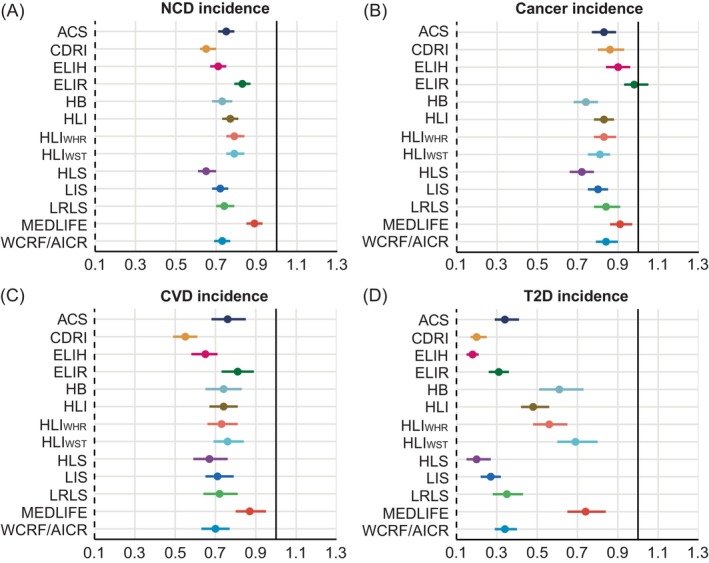
Fully‐adjusted hazard ratios and 95% confidence intervals for primary NCD‐related incidence, including (A) NCD incident, (B) cancer incidence, (C) CVD incidence, and (D) T2D incidence, comparing the highest to the lowest category of each lifestyle score. Cox proportional hazards regression adjusted for age and sex, ethnicity, region, index of multiple deprivation, household income, education, drug use, history of cancer screening, family history of diabetes, cancer, or CVD. ACS, the American Cancer Society guidelines score; CDRI, chronic disease risk index; CVD, cardiovascular disease; ELIH, empirical lifestyle pattern score for hyperinsulinemia; ELIR, empirical lifestyle pattern score for insulin resistance; HB, Health behaviors; HLI, healthy lifestyle index; HLS, healthy lifestyle score; LIS, lifestyle inflammation score; LRLS, low‐risk lifestyle score; MEDLIFE, the Mediterranean lifestyle; NCD, non‐communicable disease; T2D, type 2 diabetes; WCRF/AICR score, World Cancer Research Fund and the American Institute for Cancer Research score.

**FIGURE 2:**
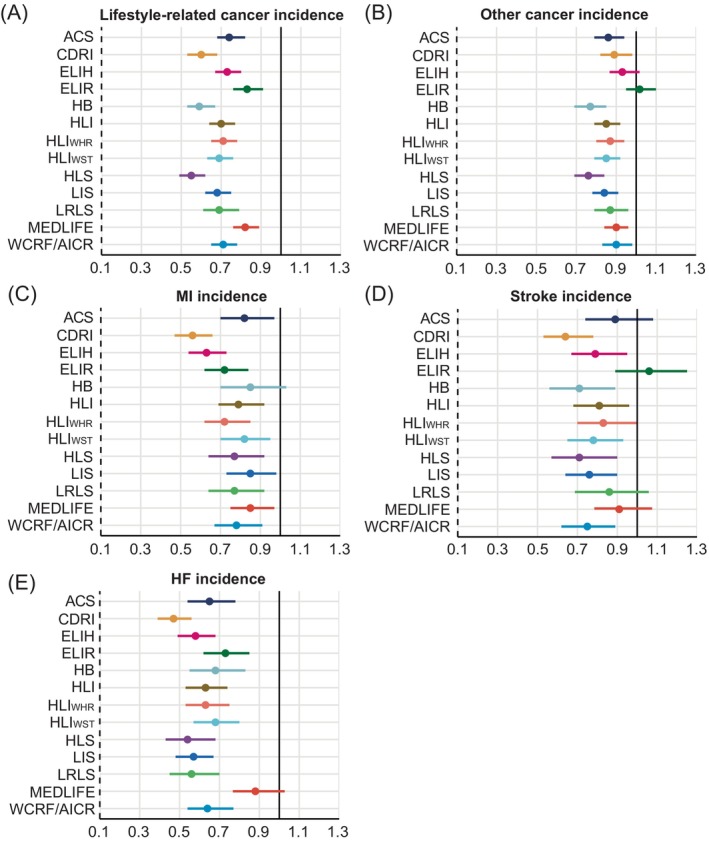
Fully‐adjusted hazard ratios and 95% confidence intervals for secondary NCD‐related incidence, including (A) lifestyle‐related cancer incidence, (B) other cancer incidence, (C) MI incidence, (D) stroke incidence, and (E) HF incidence, comparing the highest to the lowest category of each lifestyle score. Cox proportional hazards regression adjusted for age and sex, ethnicity, region, index of multiple deprivation, household income, education, drug use, history of cancer screening, family history of diabetes, cancer, or CVD. ACS, the American Cancer Society guidelines score; CDRI, chronic disease risk index; ELIH, empirical lifestyle pattern score for hyperinsulinemia; ELIR, empirical lifestyle pattern score for insulin resistance; HB, health behaviors; HLI, healthy lifestyle index; HLS, healthy lifestyle score; HF, heart failure; LIS, lifestyle inflammation score; LRLS, low‐risk lifestyle score; MEDLIFE, the Mediterranean lifestyle; MI, myocardial infarction; WCRF/AICR score, World Cancer Research Fund and the American Institute for Cancer Research score.

An overall decreasing trend was observed in the risk of incident events with higher levels for most lifestyle scores (*P* for trend <0.001) (Figures 1 and 2, Supplementary Tables 4 and 5). In the analyses of NCD incidence, all lifestyle scores exhibited a statistically significant association with incident NCDs, with HRs ranging from 0.65 to 0.89 (Figure 1A). The associations were strongest for CDRI (HR for highest versus lowest category 0.65, 95% CI 0.62–0.70), HLS (0.65, 0.61–0.70), and ELIH (0.71, 0.67–0.75).

Regarding the incidence of the specific NCDs assessed, the strongest relationships were observed with incident T2D, with HRs ranging from 0.18 to 0.74. ELIH (HR for highest versus lowest category 0.18, 95% CI 0.15–0.21), CDRI (0.20, 0.17–0.25), and HLS (0.20, 0.15–0.27) showed particularly strong associations with T2D (Figure 1D). The estimates for incident cancer (Figure 1B) were less pronounced than those for incident CVD (Figure 1C). HLS (HR for highest versus lowest category 0.72, 95% CI 0.66–0.78), HB (0.74, 0.68–0.80), and LIS (0.80, 0.75–0.85) were identified as the top three scores most strongly associated with cancer, while CDRI (0.55, 0.49–0.61), ELIH (0.65, 0.58–0.71), and HLS (0.67, 0.59–0.76) were the top three scores associated with CVD. The weakest associations were observed between MEDLIFE and the incidence of CVD and T2D, and ELIR with incident cancer.

For cancer, the associations between the lifestyle scores and lifestyle‐related cancers (HRs ranging from 0.55 to 0.83) were more pronounced compared to other types of cancer as expected (0.76–1.02) (Figure 2A and B). Regarding incident CVD‐related endpoints, risk reduction with higher scores was generally greatest for HF (HRs ranging from 0.47 to 0.88) and weakest for stroke (0.64–1.06). CDRI showed the highest association for HF and stroke incidence (HR for highest versus lowest category 0.64, 95% CI 0.53–0.78 for stroke; 0.47, 0.39–0.56 for HF) (Figure 2C–E).

### 3.3 Associations of the 13 lifestyle scores with all‐cause and NCD‐related mortality

**FIGURE 3:**
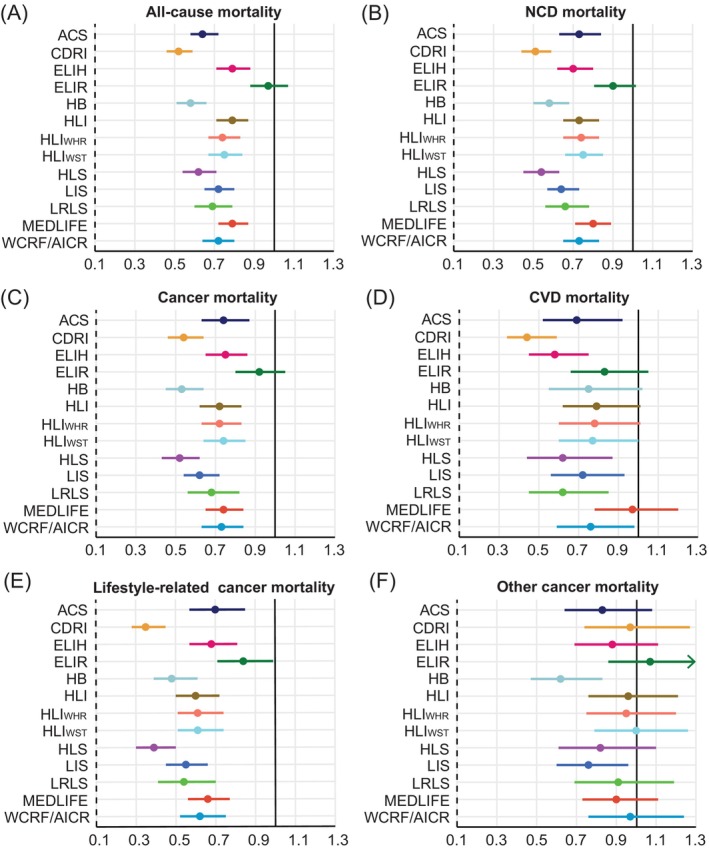
Fully‐adjusted hazard ratios and 95% confidence intervals for primary and secondary NCD‐related mortality, including (A) all‐cause mortality, (B) NCD mortality, (C) cancer mortality, (D) CVD mortality, (E) lifestyle‐related cancer mortality, and (F) other cancer mortality, comparing the highest to the lowest category of each lifestyle score. Cox proportional hazards regression adjusted for age and sex, ethnicity, region, index of multiple deprivation, household income, education, drug use, history of cancer screening, family history of diabetes, cancer, or CVD. ACS, the American Cancer Society guidelines score; CVD, cardiovascular disease; CDRI, chronic disease risk index; ELIH, empirical lifestyle pattern score for hyperinsulinemia; ELIR, empirical lifestyle pattern score for insulin resistance; HB, health behaviors; HLI, healthy lifestyle index; HLS, healthy lifestyle score; LIS, lifestyle inflammation score; LRLS, low‐risk lifestyle score; MEDLIFE, the Mediterranean lifestyle; NCD, non‐communicable disease; WCRF/AICR score, World Cancer Research Fund and the American Institute for Cancer Research score.

In general, inverse associations were observed between higher lifestyle scores and death from any cause, NCD mortality, and cancer mortality (Figure 3A–C, Supplementary Table 6). CDRI (HR for highest versus lowest category 0.52, 95% CI 0.46–0.59 for all‐cause mortality; 0.51, 0.44–0.59 for NCD mortality; 0.54, 0.46–0.64 for cancer mortality), HB (0.58, 0.51–0.66; 0.58, 0.50–0.68; 0.53, 0.45–0.64), and HLS (0.62, 0.54–0.71; 0.54, 0.45–0.63; 0.52, 0.43–0.62) were found to have the most robust associations with mortality, respectively. For total CVD mortality, the risk also showed a decreasing trend with higher scores, although 95% CIs were broad (Figure 3D). ELIR was not associated with any of these events. Concerning cancer‐related mortality, a higher lifestyle score was associated with a lower risk of lifestyle‐related cancer mortality for all 13 lifestyle scores (HRs ranging from 0.36 to 0.84), whereas most lifestyle scores showed no significant associations with other cancer mortality (Figure 3E and F, Supplementary Table 7).

### 3.4 Associations of the 13 lifestyle scores with NCD incidence and mortality in subgroups

In analyses stratified by age, sex, and deprivation index (Supplementary Figures 2 and 3, Supplementary Tables 8 and 9), associations with incident NCD were somewhat more pronounced in individuals younger than 60 years and women compared to older individuals and men. Associations with NCD mortality were also somewhat more pronounced in those younger than 60 years. No clear pattern of differential associations was observed for incidence when stratified by deprivation index, or for mortality when stratified by sex and deprivation index.

## 4 DISCUSSION

In this large‐scale prospective cohort study, we examined and compared the associations between 13 simple lifestyle scores and incidence and mortality of major NCDs. In general, a healthier combined lifestyle was associated with a lower incidence of overall NCDs, cancer, CVD, and T2D, as well as with reduced all‐cause, NCD‐related, and cancer‐related mortality, regardless of the specific NCD for which the lifestyle scores were developed. The strongest relationship with lifestyle scores was observed in the analysis of incident T2D, followed by CVD and cancer. The top three lifestyle scores for predicting incident NCDs were CDRI, ELIH, and HLS, while for NCD mortality, the leading scores were CDRI, HB, and HLS.

The assessed lifestyle scores were not only associated with the diseases they were developed for but also proved useful in predicting other NCDs and overall NCDs, supporting our hypothesis of extended utility for multiple NCDs. The ACS and WCRF/AICR scores were developed to assess how adherence to cancer prevention guidelines was linked to cancer‐related outcomes.^16^, ^17^ Our results were in line with Greenlee et al. who reported that participants with higher points on ACS also had a 34% lower risk of developing CVD and a 40% lower risk of CVD mortality compared with those with the lowest score.^18^ Similar to our results, the Diabetes Prevention Program Outcomes Study found a strong association of the WCRF/AICR score with T2D incidence.^19^ ELIH and ELIR were constructed to reflect long‐term insulin exposure and overall insulin resistance by a lifestyle with insulinemic potential.^20^ Previous work also showed that the ELIH was associated with colorectal and digestive system cancers, and ELIR with liver cancer, colorectal cancer, and CVD.^21^, ^22^, ^23^, ^24^ Our findings regarding the ELIH and ELIR scores were consistent; yet, we additionally found that higher scores on ELIH were strongly associated with lower CVD incidence. These associations suggest that different health risk factors have overlapping effects across multiple NCDs. Thus, despite uncertainty about the mechanism, our results highlight that cancer, CVD, and T2D can be prevented altogether by adopting a common healthy lifestyle. These results will be of value to policymakers, researchers, and the general public. In the current era of exploding health information, our results should encourage cancer, CVD, and T2D‐related organizations to jointly create a unified lifestyle guideline to promote primary prevention and early detection of multiple NCDs.

The lifestyle scores were not equally good in our benchmark analysis. The associations of each score differed by outcome. For cancer‐related outcomes, the ELIH, ELIR, MEDLIFE, and WCRF/AICR show relatively weak correlations, and all of them do not include smoking in their scores. In contrast, HLS, HB, and LIS scores included smoking and showed the strongest associations with cancer. This discrepancy may be because smoking has the greatest impact on overall cancer risk compared to other lifestyle factors.^25^ The definition of smoking in the lifestyle scores also seemed to affect the associations. The HLS not only includes current smoking but also takes into account previous heavy smoking, and showed the strongest associations with cancer outcomes.^26^ Similarly, the HLI, which includes former long‐term smoking as a risk factor, was also strongly associated with cancer risk in our study. Although the CDRI and LRLS also consider the risk of past smoking, they did not perform as well as the top‐performing scores, possibly because they defined all past smokers as the same category regardless of the amount of smoking and duration. The LIS was constructed by assigning weights to lifestyle factors based on their association with a panel of circulating biomarkers of inflammation.^27^ Based on the underlying biological pathways of chronic inflammation strongly associated with cancer development,^28^ the performance of the LIS suggests that inflammation‐related lifestyle factors are also effective predictors of cancer risk.

For CVD, the MEDLIFE score exhibited the worst performance. MEDLIFE incorporates unique lifestyle habits such as napping, sleep duration, and the Mediterranean diet, among others, but excludes smoking and BMI. Given that both are well‐established contributors to CVD risk,^29^ omitting these key factors might substantially weaken the predictive validity of MEDLIFE. The ELIR also showed a relatively weak association with CVD, which was created to identify a lifestyle pattern predictive of insulin resistance, while the ELIH designed to reflect hyperinsulinemia demonstrated a strong association. Our findings are consistent with previous studies that reported hyperinsulinemia and insulin resistance as risk factors for CVD,^30^, ^31^ although the role of hyperinsulinemia and insulin resistance as a cardiovascular risk factors remains controversial. Notably, our study revealed ELIH as a stronger predictor for CVD risk than ELIR. CDRI showed particularly strong associations with both incidence and mortality of CVD. CDRI was created according to recommendations on chronic disease prevention from the National Research Council and the US Department of Health and Human Service.^32^ CDRI includes smoking and BMI but does not include physical activity. Further analysis is required to determine which specific component contributes most significantly to its stronger association with CVD. Our findings showed the LIS with a good performance for CVD risk, aligning with studies indicating that inflammation increases the risk of CVD.^33^

For T2D, the results were weakest for HB and MEDLIFE, both of which do not include BMI. The important role of high BMI in T2D risk may explain their limited predictive performance. Although multiple studies from various countries showed that central obesity indices were considered to have a stronger association with T2D than BMI,^34^, ^35^ our results on the HLI with different anthropometric measures suggest that BMI is a useful and easy‐to‐assess component of a lifestyle score to predict the risk of T2D. Both the ELIH and ELIR were strongly associated with T2D risk, although these two indices do not include smoking status, possibly because obesity exerts a more dominant influence on T2D development than smoking.^36^, ^37^ The LIS in the study was also strongly related to T2D, supporting evidence linking chronic low‐grade inflammation to T2D pathogenesis.^38^ Among the best‐performing indices (CDRI, HLS, WCRF/AICR, and ACS), all included BMI. When synthesizing the patterns observed across all three NCDs, the HLS emerged as the strongest predictor of NCDs. This is likely due to its inclusion of the five most important modifiable risk factors and its consideration of the cumulative impact of past heavy smoking behavior.

Collectively, a healthier lifestyle was most strongly associated with risk reduction of T2D and less strongly with total cancer. Cancer may be less dependent on behavior than T2D. A multinational cohort study reported a strong inverse association between adherence to a healthy lifestyle and risk of T2D, with a 33% risk reduction, whereas the association with cancer risk was weaker, showing only an 11% reduction.^39^ Furthermore, the lower effectiveness may be due to certain cancer types having a weaker association with lifestyle factors, which attenuates the overall impact on total cancer incidence. This assumption is supported by our subgroup analyses on lifestyle‐related cancers. Because of the heterogeneity of various cancers, the associations of lifestyle scores and the reduction of risk depend on the specific type of cancer. Given that there is no conclusive evidence on which cancers are affected by lifestyles and to what extent they are affected, further research is needed.

In line with our results in the other subgroup analysis, numerous studies have revealed that women and younger individuals were more likely to benefit from healthy lifestyle behaviors. The inverse association between specific factors comprising lifestyle scores and NCDs supported our results. Wang et al. showed various dietary patterns influenced more on women or people younger than 65 years in terms of developing major NCD.^40^ A review from Bolego et al. summarized that smoking posed a stronger risk factor for coronary heart disease in women who were current smokers compared to men.^41^ Moreover, drinking three bottles of wine per week was associated with an absolute risk of cancer of 1.9% for males, but 3.6% for females over their lifetime.^42^ However, there remains insufficient evidence to derive definitive conclusions. In a 6‐year prospective study, Yoo et al. indicated that a higher level of alcohol drinking would result in more alcohol‐related cancers and total cancer in individuals ≥65 years.^43^

This study is the first to directly compare existing simple lifestyle scores side‐by‐side within the same population regarding their ability to predict incidence and mortality of major NCDs beyond their original field of application. Using NCDs as a composite endpoint extends knowledge about the preventive potential of the assessed lifestyle scores and enables the selection of the scores best suited for widespread use. Further strengths of this study are its large sample size, the prospective design, and the long‐term follow‐up period available for the UK Biobank cohort.

Several limitations of this study should be acknowledged. First, for certain lifestyle scores, we lacked adequate information in the UK Biobank to replicate them exactly as originally developed. Wherever possible, variables were replaced with alternatives available. Still, such modifications in the construction of a lifestyle score may affect the strength of the associations. Second, for lifestyle scores identified by self‐reported 24‐h dietary assessment, random error caused by day‐to‐day variability and systematic error related to recall bias or misreporting of food intake would impact the results. To improve accuracy, we relied on at least two times of 24‐h dietary assessments and excluded implausible energy intake to improve representativity for typical dietary intake, and to account for seasonal variations in dietary intake. At the same time, online 24‐h dietary assessment had some benefits. It reduced recall bias to some extent by asking about yesterday's diet, and online inquiry may make the participants more honest. Third, differences in participant characteristics by response pattern could limit generalizability. In UK Biobank, participants who responded to the online questionnaire were more likely to be female, older, white, less poor, and more educated than those who did not respond. Participants who responded multiple times were more likely to be white, older, and more educated than those who completed only one response.36 Fourth, early symptoms of T2D are often overlooked, leading to failure to obtain a timely diagnosis, so the time variable can easily be calculated longer than the real time from baseline to T2D diagnosis. To minimize this bias, we used both hospital inpatient admission data and primary care data to identify incident T2D cases. Lastly, although we made efforts to divide the population into four equally sized groups based on each score, the non‐continuous nature of the scores made it challenging to ensure even distribution, which may affect the fairness of the head‐to‐head comparison.

## 5 CONCLUSIONS

Our results suggest that the use of simple lifestyle scores designed for a single disease group can be extended for predicting multiple NCDs and mortality. The inclusion of BMI and smoking in lifestyle scores is crucial. We found that the HLS and CDRI were the overall best performing scores to predict NCD risk and mortality, which might be useful information for future investigations and assessments. These findings highlight the value of collaborative efforts among organizations related to cancer, CVD, and T2D to develop common lifestyle recommendations to reduce the burden of NCDs.

## AUTHOR CONTRIBUTIONS

**Jie Ding:** Methodology; software; investigation; conceptualization; writing – original draft; writing – review and editing; formal analysis; data curation; visualization. **Ben Schöttker:** Resources; writing – review and editing. **Hermann Brenner:** Methodology; writing – review and editing; supervision. **Michael Hoffmeister:** Conceptualization; methodology; supervision; writing – review and editing.

## FUNDING INFORMATION

The UK Biobank was established by the Wellcome Trust, Medical Research Council, Department of Health, Scottish government, and Northwest Regional Development Agency. It has also received funding from the Welsh assembly government, British Heart Foundation, Cancer Research UK, and Diabetes UK. UK Biobank is supported by the National Health Service (NHS). This project was supported by a grant from the German Ministry for Education and Research (Grant No. 01KD2104A). JD was supported by the Chinese Scholarship Council (CSC, No. 202006180030). The sponsors had no role in the design of the study or acquisition of data, nor any other role in the publication of this analysis.

## CONFLICT OF INTEREST STATEMENT

All authors declare no potential conflicts of interest.

## Supporting information


**Data S1.** Supporting Information.

## Data Availability

This work has been conducted using the UK Biobank Resource under Application Number “66591”. The UK Biobank is an open access resource and bona fide researchers can apply to use the UK Biobank dataset by registering and applying at https://www.ukbiobank.ac.uk/. Further information is available from the corresponding author upon request.
