# Supplementary material for: Thirteen simple lifestyle scores and risk of cancer, cardiovascular disease, diabetes, and mortality: Prospective cohort study in the UK Biobank
Source: Int J Cancer. 2025 Jul 24;157(12):2495–505. doi: 10.1002/ijc.70064 (PMC12541561; doi:10.1002/ijc.70064)
Supplement: Supplementary file 1 — Data S1. Supporting Information. [file IJC-157-2495-s001.pdf]

Supplementary Material

Thirteen simple lifestyle scores and risk of cancer, cardiovascular disease, diabetes, and mortality: prospective cohort study in the UK Biobank

Jie Ding, Ben Schöttker, Hermann Brenner, Michael Hoffmeister

Table of contents

Supplementary Table 1. Scoring details of the 13 lifestyle scores 1

Supplementary Methods 4

Supplementary Table 2. Outcome definitions 5

Supplementary Figure 1. Flow chart of the study exclusions 6

Supplementary Table 3. Baseline Characteristics of the Study Population 7

Supplementary Table 4. Associations of 13 lifestyle scores with primary NCD-related incidence outcomes 8

Supplementary Table 5. Associations of 13 lifestyle scores with secondary NCD-related outcomes incidence 10

Supplementary Table 6. Associations of 13 lifestyle scores with primary NCD-related outcomes mortality 12

Supplementary Table 7. Associations of 13 lifestyle scores with secondary NCD-related outcomes mortality 14

Supplementary Figure 2. Fully-adjusted hazard ratios and 95% confidence intervals for major NCD incidence in subgroups 16

Supplementary Figure 3. Fully-adjusted hazard ratios and 95% confidence intervals for major NCD mortality in subgroups 17

Supplementary Table 8. Associations of 13 lifestyle scores with major NCD incidence in subgroups 18

Supplementary Table 9. Associations of 13 lifestyle scores with major NCD mortality in subgroups 20

References 22

Supplementary Table 1. Scoring details of the 13 lifestyle scores

Lifestyle score	Smoking	Alcohol consumption	Diet	Physical activity	BMI	Other components	Scoring system	Total points
ACS[1]	N/A	Daily or almost daily; ≤2 times/wk; special occasions only or never	0-2; 3-6; 7-9 points (a point if ≥5 servings/d fruits and vegetables, an additional 1 or 2 points for being in the 2nd or 3rd tertile of unique fruits and vegetables consumed per month; daily servings of whole grains/daily servings of whole grains plus refined grains: 0 to 3 for the quartile; 0 to 3 for the quartile distribution of processed and red meats consumed servings per day)	<8.75; 8.75 to <17.5; ≥17.5 MET-h/wk	>30; 25-<30; <25 (kg/m^2)	N/A	0, 1, 2	8
CDRI[2]	Never-smoker; ex-smoker; current smoker ≤1ppd; current smoker 1.1-1.5 ppd; current smoker >1.5 ppd	Men: 1-7 (drinks/wk); all others; Women: 1-3 (drinks/wk); all others	Fat intake from animal products >55 g/day; all others; Fruit and vegetable consumption >4 servings/day; all others	N/A	Men: 19.6-24.8; <19.6 or 24.9-29.2; 29.3-32.5; ≥32.6; women: 18.5-23.6; <18.5 or 23.7-30.3; 30.4-35.9; ≥36.0 (kg/m^2)	N/A	0, 1, 2, 3, 4	10
ELIH[3]	N/A	Liquor*0.072, wine* −0.071	Positive associations: margarine*0.041, butter* 0.058*, red meat*0.089, fruit juice* 0.042, inverse associations, coffee*−0.020, whole fruit* −0.029, high-fat dairy products* −0.054, snacks*−0.024	Average MET-h/wk *−0.001	BMI*0.051	N/A	Coefficient weights	N/A
ELIR[3]	N/A	Liquor*−0.122, wine*−0.171	Positive associations: refined grains*0.076, red meat*0.181, margarine*0.099, tomatoes*0.135, low-energy beverages*0.051, fruit juice*0.068, potatoes*0.160, processed meat*0.124, other vegetables*0.070, and tea*0.027; inverse associations: coffee*-0.041, high-fat dairy products*−0.064, green leafy vegetables*−0.064	Average MET-h/wk *−0.001	BMI*0.047	N/A	Coefficient weights	N/A
HB[4]	Non-smoker	<14 units/wk (1 unit g alcohol)	Fruit and vegetable intake ≥5 (servings/d)	Not inactive (≥30min/d)	N/A	N/A	Binary	4

Lifestyle score	Smoking	Alcohol consumption	Diet	Physical activity	BMI	Other components	Scoring system	Total points
HLI[5]	Never, ex-smokers quit>10, ex-smokers quit≤10 (years), current≤15, current>15 (cigarettes/d)	<6, 6-<12, 12-<24, 24-<60, ≥60 (g/d)	Quintile of healthy diet score: fruits and vegetables, cereal fiber, red and processed meat, the ratio of polyunsaturated to saturated fat, trans-fats, and glycemic load	MET-h/wk quintile values	<22, 22-23.9, 24-25.9, 26-29.9, ≥30 (kg/m^2)	N/A	4, 3, 2, 1, 0	20
HLI_{WHR}[5]	Never, ex-smokers quit>10, ex-smokers quit≤10 (years), current≤15, current>15 (cigarettes/d)	<6, 6-<12, 12-<24, 24-<60, ≥60 (g/d)	Quintile of healthy diet score: fruits and vegetables, grains, red and processed meat, the ratio of polyunsaturated to saturated fat, trans-fats, and glycemic load	MET-h/wk quintile values	N/A	WHR: quintile	4, 3, 2, 1, 0	20
HLI_{WST}[5 6]	Never, ex-smokers quit>10, ex-smokers quit≤10 (years), current≤15, current>15 (cigarettes/d)	<6, 6-<12, 12-<24, 24-<60, ≥60 (g/d)	Quintile of healthy diet score: fruits and vegetables, grains, red and processed meat, the ratio of polyunsaturated to saturated fat, trans-fats, and glycemic load	MET-h/wk quintile values	N/A	WST: < 80, 80-88, and ≥ 88 (cm)	WST: 2, 1, 0; others: 4, 3, 2, 1, 0	18
HLS[7]	Never smoker, or former smoker (<30 pack years)	Women: ≤12, men≤24 (g/day)	Red and processed meat 0, ≤1,1, 2-6, ≥7 (times/wk), vegetables <1, 1, ≥2 (tablespoons/day), fruit 0, 1, >1 (piece/d), cheese 0, ≤1, 1, 2-6, ≥7 (times/wk), wholegrains 0, <1, 1, <1 and <7, 7, >7 (times/wk), fish 0, ≤1, 1, 2-6, ≥7 (times/wk)	500 (MET minutes/wk)	18.5-25 (kg/m^2)	N/A	Red and process ed meat 0, 1, 2, 3, 4 Cheese/ Wholegr ains 10, 8, 6, 4, 2, 0 Fish 4, 3, 2, 1, 0 Fruit/ve getables 4, 2, 0 Others binary	

Lifestyle score	Smoking	Alcohol consumption	Diet	Physical activity	BMI	Other components	Scoring system	Total points
LIS[8]	Current smoker=0.50; did not currently smoke tobacco =0	> 14 for women, > 28 for men=0.3; 14 for women, 14–28 (g/d) for men=-0.66; nondrinker=0	N/A	Vigorous or moderate activity ≥ 4 = -0.41; 1–3 = -0.18; 0 (times/wk) =0	<25=0; 25-29.99 =0.89; ≥30 (kg/m²) =1.57	N/A	Coefficient weights	N/A
LRLS[9]	Never smoking	Men: 5-30, women: 5-15 (g/d)	AHEI score in the top 40%*: vegetables 0-≥5, fruit 0-≥4, nuts 0-≥1, red and processed meats≥1.5-0, sugar-sweetened beverages ≥1-0, trans fat ≥4-≤0.5 (servings/d), sodium, whole grains women 0-75 men 0-90, omega-6 fatty acids 0-250 (g/d)	>30 (min/d) of moderate or vigorous activities	18.5-24.9 (kg/m²)	N/A	Binary	5
MEDLIFE[10]	N/A	Wine: women: ≤0.5, men: ≤1 (serving/d)	Mediterranean food consumption: red meat <2, processed meat<2, white meat= 2-4, fish/seafood≥2, fruit 3–6, vegetables≥2, cereals 3–6, water ≥6 (servings/d), limit salt at meals (Yes), preference for whole grain products (Yes)	>300 min/wk	N/A	Nap (sometimes or usually), hours of sleep 6–8 h/d, watching TV≤2h/d, frequency of friends/family visits (about or more than 1/every few months), group activity (Yes)	Binary	4
WCRF /AICR[11]	N/A	Men: >28, >0–≤28, 0<br>Women: >14, >0–≤14, 0 (g/day)	Sugar-sweetened drinks: >250, >0–≤250, 0<br>red meat >500 or processed meat ≥100, red meat ≤500 and processed meat 21–<100, red meat ≤500 and processed meat <21<br>tertile of energy density estimated food nutrients<br>fruits and vegetables: <200, 200–<400, ≥400 (g/day)<br>total fiber: <15, 15–<30, ≥30 (g/day)	Moderate-to-vigorous physical activity <75, 75–<150, ≥150 (min/wk)	<18.5 or ≥30, 25-30, 18.5-25 (kg/m²)	WST<br>Men: ≥102, 94–<102, <94<br>Women: ≥88, 80–<88, <80 (cm)	0; 0.5; 1 or 0; 0.25; 1	7

Abbreviations: ACS, the American Cancer Society guidelines score; CDRI, chronic disease risk index; ELIH, empirical lifestyle pattern score for hyperinsulinemia; ELIR, empirical lifestyle pattern score for insulin resistance; HB, Health behaviors; HLI, healthy lifestyle index; HLS, healthy lifestyle score; LIS, lifestyle inflammation score; LRLS, low-risk lifestyle score; MEDLIFE, the Mediterranean lifestyle; WCRF/AICR score, World Cancer Research Fund and the American Institute for Cancer Research score; BMI, body mass index; WST, waist circumference; WHR, waist-to-hip ratio.

Supplementary Methods

Dietary assessment

For HLS and MEDLIFE, the dietary information was derived from a food frequency questionnaire filled out at the assessment center at baseline. For the other 11 scores, information on the quantity of dietary intake was obtained using a dietary questionnaire (Oxford WebQ), based on a 24-hour dietary recall of the previous day. The questionnaire was implemented towards the end of the recruitment phase at the assessment center. Subsequently, four additional cycles of data collection were repeated by sending e-mails to participants at 3-4 monthly intervals (cycle 1: February 2011 to April 2011; cycle 2: June 2011 to September 2011; cycle 3: October 2011 to December 2011; cycle 4: April 2012 to June 2012). Mean values were calculated from ≥ two 24-hour dietary assessments to minimize the effects of random error and within-person variability.[12 13]

Ascertainment of outcomes

Prevalent cases of cancer and CVD were identified by self-report, cancer registries, and hospital admission records at baseline. Incident cases of cancer and CVD were defined as the first diagnosis in cancer registries, hospital admission, or death records. Prevalent cases of diabetes were identified by data from self-reports, primary care, hospital inpatient records, medication, and biomarkers (HbA_{1c} ≥48 mmol/mol) at baseline, while incident T2D was defined as the first diagnosis recorded on the primary care, hospital admission, and death database.[14] Outcomes were classified using ICD-9 and ICD-10 (international classification of diseases, ninth and tenth revisions, respectively) codes. The following hospital admission records were linked to the datasets: the Scottish Morbidity Records (Scotland), the Patient Episode Database for Wales (Wales), and the Health Episode Statistics (England). Death records were obtained from the National Health Service (NHS) (England and Wales) and the NHS Central Register Scotland (Scotland). Primary care data origin from various data suppliers (TPP, Vision, EMIS).

Supplementary Table 2. Outcome definitions

Outcomes	Self-reports	ICD-10 code	ICD-9 code	Medication	Biomarkers
Incidence and mortality of cancer	1001-1059, 1063-99999	C00-C43, C45-C97	140-172, 174-239	N/A	N/A
Incidence and mortality of lifestyle-related cancer (breast cancer, kidney cancer, endometrial cancer, ovarian cancer, lung cancer, digestive system cancer (esophagus, stomach, small intestine, colon, rectum, anus and anal canal, pancreas, gallbladder, and liver))	1001, 1002, 1017-1026, 1027, 1028, 1034, 1039, 1040	C15.X-C26.X, C33, C34.X, C50.X, C54.1, C56, C64, C65	150-159, 174.X, 1890, 1820, 1830, 1623, 1629	N/A	N/A
Incidence and mortality of T2D	1220, 1221, 1222, 1223	E10.X-E14.X	250.X	Insulin product (1140883066), metformin (1140884600), metformin/rosiglitazone (1141189090), glimepiride (1141152590), gliclazide (1140874744), glibenclamide (1140874718), amaryl (1141156984), acarbose (1140868902), pioglitazone (1141171646), nateglinide (1141173882), repaglinide (1141168660)	HbA1c (30750)
Incidence of CVD	1491, 1583, 1075, 1076, 1081, 1086	N/A	N/A	N/A	N/A
Mortality of CVD	N/A	I00.X-I99.X	N/A	N/A	N/A
Myocardial infarction	1075	I21.X, I22.X, I23.X, I25.2	410.X, 411.X, 412.X	N/A	N/A
Heart failure	1076	I50.X, I11.0, I13.0, I13.2	428.X	N/A	N/A
Stroke	1491, 1583, 1081, 1086	I60.X, I61.X, I63.X, I64.X, I62.9, I69.0, I69.1, I69.3, I69.4	430.X, 431.X, 434.X, 436.X	N/A	N/A

Prevalent cases were identified by self-reports, ICD-9, ICD-10, medication, and biomarkers code, while incident cases were identified by ICD-10 code.

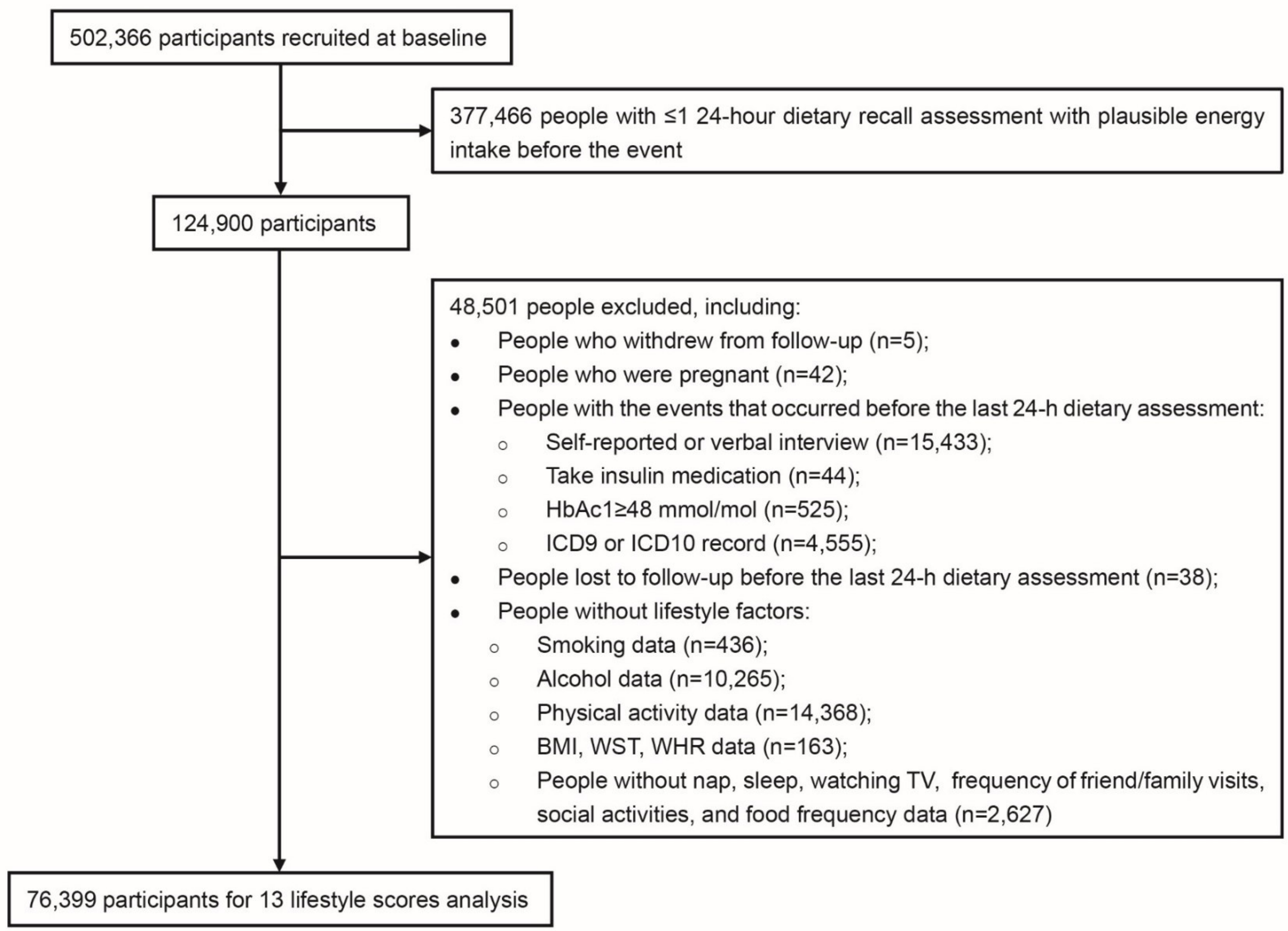

Supplementary Figure 1. Flow chart of the study exclusions

Abbreviations: BMI, body mass index; WST, waist circumference; WHR, waist-to-hip ratio.

Supplementary Table 3. Baseline Characteristics of the Study Population

Characteristic	Number	Percentage (%)
**Age**	55.5 (mean)	7.8 (SD)
**Female**	40,136	52.5
**Ethnicity**		
White	74,010	96.9
Other ethnic groups	2,205	2.9
Unknown	186	0.2
**Education**		
High degree	50,337	65.9
Middle degree	22,275	29.2
Others	3,678	4.8
Unknown	111	0.1
**Household income**		
Less than £18 000	7,952	10.4
£18 000–£51 999	36,199	47.4
Greater than £52 000	26,892	35.2
Unknown	5,358	7.0
**Index of multiple deprivation (quintile)**		
1	14,863	19.5
3	14,838	19.5
5	14,872	19.5
Unknown	1,992	2.6
**Lifestyle**		
Current smoker	5,094	6.7
BMI (Kg/m^2)	26.3 (mean)	4.2 (SD)
WST (cm)	87.9 (mean)	12.6 (SD)
WHR	0.86 (mean)	0.1 (SD)
Alcohol (g/d)	23.4 (mean)	23.8 (SD)
Physical activity (MET-min/week)	2,435.5 (mean)	2,307.4 (SD)
Energy intake (kJ/d)	8,730.4 (mean)	2,687.2 (SD)
**Family history of diseases**		
Cancer	26,318	34.4
CVD	42,506	55.6
T2D	14,597	19.1
Unknown	1,042	1.4

Values are means (SD) for continuous variables and percentages for categorical variables if not specified otherwise. Abbreviations: SD, standard deviation; WST, waist circumference; WHR, waist-to-height ratio; MET, Metabolic Equivalent of Task.

Supplementary Table 4. Associations of 13 lifestyle scores with primary NCD-related incidence outcomes

Lifestyle score	Category	NCD incidence		Cancer incidence		CVD incidence		T2D incidence	
		**HR (95% CI)**	P_{trend}	**HR (95% CI)**	P_{trend}	**HR (95% CI)**	P_{trend}	**HR (95% CI)**	P_{trend}
Cases/person-years		12,214/748,391		8,334/764,802		3,535/786,858		1,698/792,878	
ACS	C1	1.00	<0.001	1.00	<0.001	1.00	<0.001	1.00	<0.001
	C2	0.90 (0.86, 0.95)		0.97 (0.92, 1.03)		0.91 (0.83, 0.99)		0.61 (0.54, 0.68)	
	C3	0.84 (0.80, 0.88)		0.92 (0.87, 0.98)		0.86 (0.79, 0.94)		0.43 (0.37, 0.49)	
	C4	0.75 (0.71, 0.79)		0.83 (0.77, 0.89)		0.76 (0.68, 0.85)		0.34 (0.29, 0.41)	
CDRI	C1	1.00	<0.001	1.00	<0.001	1.00	<0.001	1.00	<0.001
	C2	0.77 (0.73, 0.81)		0.91 (0.85, 0.98)		0.69 (0.63, 0.76)		0.50 (0.44, 0.56)	
	C3	0.67 (0.63, 0.70)		0.83 (0.78, 0.89)		0.57 (0.52, 0.63)		0.32 (0.28, 0.36)	
	C4	0.65 (0.62, 0.70)		0.86 (0.80, 0.93)		0.55 (0.49, 0.61)		0.20 (0.17, 0.25)	
ELIH	C1	1.00	<0.001	1.00	<0.001	1.00	<0.001	1.00	<0.001
	C2	0.81 (0.77, 0.85)		0.96 (0.91, 1.02)		0.78 (0.72, 0.85)		0.42 (0.37, 0.47)	
	C3	0.70 (0.67, 0.74)		0.87 (0.82, 0.92)		0.69 (0.63, 0.76)		0.24 (0.21, 0.28)	
	C4	0.71 (0.67, 0.75)		0.90 (0.84, 0.96)		0.65 (0.58, 0.71)		0.18 (0.15, 0.21)	
ELIR	C1	1.00	<0.001	1.00	0.368	1.00	<0.001	1.00	<0.001
	C2	0.86 (0.82, 0.90)		0.95 (0.89, 1.01)		0.88 (0.80, 0.95)		0.55 (0.49, 0.61)	
	C3	0.79 (0.75, 0.83)		0.94 (0.89, 1.00)		0.76 (0.69, 0.83)		0.38 (0.33, 0.43)	
	C4	0.83 (0.79, 0.87)		0.98 (0.93, 1.05)		0.81 (0.73, 0.89)		0.31 (0.26, 0.36)	
HB	C1	1.00	<0.001	1.00	<0.001	1.00	<0.001	1.00	<0.001
	C2	0.86 (0.80, 0.91)		0.86 (0.79, 0.93)		0.81 (0.72, 0.92)		0.82 (0.69, 0.97)	
	C3	0.79 (0.74, 0.84)		0.81 (0.75, 0.88)		0.74 (0.66, 0.83)		0.70 (0.60, 0.82)	
	C4	0.73 (0.68, 0.78)		0.74 (0.68, 0.80)		0.74 (0.65, 0.83)		0.61 (0.51, 0.73)	
HLI	C1	1.00	<0.001	1.00	<0.001	1.00	<0.001	1.00	<0.001
	C2	0.89 (0.85, 0.93)		0.95 (0.89, 1.00)		0.80 (0.73, 0.87)		0.72 (0.64, 0.81)	
	C3	0.84 (0.80, 0.88)		0.89 (0.84, 0.95)		0.77 (0.70, 0.84)		0.62 (0.54, 0.71)	
	C4	0.77 (0.73, 0.81)		0.83 (0.78, 0.88)		0.74 (0.67, 0.81)		0.48 (0.42, 0.56)	
HLI$_{WHR}$	C1	1.00	<0.001	1.00	<0.001	1.00	<0.001	1.00	<0.001
	C2	0.90 (0.86, 0.94)		0.92 (0.87, 0.98)		0.85 (0.77, 0.92)		0.78 (0.69, 0.89)	
	C3	0.85 (0.81, 0.89)		0.86 (0.81, 0.91)		0.84 (0.77, 0.93)		0.73 (0.64, 0.84)	
	C4	0.79 (0.75, 0.84)		0.83 (0.78, 0.89)		0.73 (0.66, 0.81)		0.56 (0.48, 0.65)	
HLI$_{WST}$	C1	1.00	<0.001	1.00	<0.001	1.00	<0.001	1.00	<0.001
	C2	0.86 (0.82, 0.90)		0.86 (0.81, 0.91)		0.84 (0.77, 0.91)		0.78 (0.68, 0.88)	
	C3	0.86 (0.82, 0.90)		0.87 (0.82, 0.92)		0.81 (0.74, 0.89)		0.77 (0.68, 0.88)	
	C4	0.79 (0.75, 0.84)		0.81 (0.75, 0.86)		0.76 (0.69, 0.84)		0.69 (0.60, 0.80)	
	C1	1.00	<0.001	1.00	<0.001	1.00	<0.001	1.00	<0.001

Lifestyle score	Category	NCD incidence		Cancer incidence		CVD incidence		T2D incidence	
		HR (95% CI)	P_{trend}	HR (95% CI)	P_{trend}	HR (95% CI)	P_{trend}	HR (95% CI)	P_{trend}
HLS	C2	0.84 (0.80, 0.88)		0.88 (0.83, 0.93)		0.84 (0.77, 0.91)		0.67 (0.60, 0.75)	
	C3	0.77 (0.73, 0.81)		0.81 (0.77, 0.86)		0.75 (0.69, 0.82)		0.53 (0.46, 0.60)	
	C4	0.65 (0.61, 0.70)		0.72 (0.66, 0.78)		0.67 (0.59, 0.76)		0.20 (0.15, 0.27)	
LIS	C1	1.00	<0.001	1.00	<0.001	1.00	<0.001	1.00	<0.001
	C2	1.00 (0.95, 1.05)		1.00 (0.94, 1.07)		0.95 (0.86, 1.04)		1.05 (0.94, 1.18)	
	C3	0.78 (0.74, 0.81)		0.86 (0.81, 0.91)		0.78 (0.71, 0.84)		0.44 (0.38, 0.50)	
	C4	0.72 (0.68, 0.76)		0.80 (0.75, 0.85)		0.71 (0.65, 0.79)		0.27 (0.22, 0.32)	
LRLS	C1	1.00	<0.001	1.00	<0.001	1.00	<0.001	1.00	<0.001
	C2	0.91 (0.87, 0.95)		0.93 (0.88, 0.99)		0.90 (0.83, 0.98)		0.82 (0.74, 0.93)	
	C3	0.82 (0.78, 0.86)		0.85 (0.80, 0.90)		0.82 (0.75, 0.90)		0.60 (0.52, 0.68)	
	C4	0.74 (0.70, 0.79)		0.84 (0.78, 0.91)		0.72 (0.64, 0.81)		0.35 (0.28, 0.43)	
MEDLIFE	C1	1.00	<0.001	1.00	0.002	1.00	0.002	1.00	<0.001
	C2	0.97 (0.93, 1.02)		0.99 (0.93, 1.05)		0.92 (0.84, 1.01)		0.89 (0.78, 1.01)	
	C3	0.96 (0.92, 1.02)		0.99 (0.93, 1.06)		0.92 (0.83, 1.01)		0.81 (0.70, 0.92)	
	C4	0.89 (0.85, 0.93)		0.91 (0.86, 0.97)		0.87 (0.80, 0.95)		0.74 (0.65, 0.84)	
WCRF/AICR	C1	1.00	<0.001	1.00	<0.001	1.00	<0.001	1.00	<0.001
	C2	0.87 (0.82, 0.91)		0.95 (0.89, 1.01)		0.83 (0.76, 0.91)		0.61 (0.54, 0.69)	
	C3	0.81 (0.78, 0.85)		0.91 (0.86, 0.97)		0.78 (0.72, 0.85)		0.50 (0.44, 0.57)	
	C4	0.73 (0.69, 0.77)		0.84 (0.79, 0.90)		0.70 (0.63, 0.77)		0.34 (0.29, 0.40)	

Cox proportional hazards regression adjusted for age and sex, ethnicity, region, index of multiple deprivation, household income, education, middle degree, drug use, history of cancer screening, family history of diabetes, cancer, or CVD. The variable used for stratification was not adjusted for. Abbreviations: NCD, non-communicable disease; ACS, the American Cancer Society guidelines score; CDRI, chronic disease risk index; ELIH, empirical lifestyle pattern score for hyperinsulinemia; ELIR, empirical lifestyle pattern score for insulin resistance; HB, Health behaviors; HLI, healthy lifestyle index; HLS, healthy lifestyle score; LIS, lifestyle inflammation score; LRLS, low-risk lifestyle score; MEDLIFE, the Mediterranean lifestyle; WCRF/AICR score, World Cancer Research Fund and the American Institute for Cancer Research score.

Supplementary Table 5. Associations of 13 lifestyle scores with secondary NCD-related outcomes incidence

Lifestyle score	Category	Lifestyle-related cancer incidence		Other cancer incidence		MI incidence		Stroke incidence		HF incidence	
		**HR (95% CI)**	P_{trend}	**HR (95% CI)**	P_{trend}	**HR (95% CI)**	P_{trend}	**HR (95% CI)**	P_{trend}	**HR (95% CI)**	P_{trend}
Cases/person-years		4,148/783,657		5,620/779,997		1,574/793,208		1,123/795,931		1,250/795,890	
ACS	C1	1.00		1.00		1.00		1.00		1.00	
	C2	0.91 (0.83, 0.98)	<0.001	0.99 (0.93, 1.07)	<0.001	0.91 (0.80, 1.04)	0.032	1.02 (0.87, 1.19)	0.389	0.84 (0.73, 0.97)	<0.001
	C3	0.84 (0.78, 0.91)		0.94 (0.87, 1.00)		0.93 (0.81, 1.06)		1.03 (0.88, 1.20)		0.71 (0.61, 0.82)	
	C4	0.74 (0.68, 0.82)		0.86 (0.79, 0.94)		0.82 (0.70, 0.97)		0.89 (0.74, 1.08)		0.65 (0.54, 0.78)	
CDRI	C1	1.00		1.00		1.00		1.00		1.00	
	C2	0.80 (0.73, 0.88)	<0.001	0.94 (0.87, 1.02)	<0.001	0.78 (0.68, 0.90)	<0.001	0.72 (0.60, 0.85)	<0.001	0.65 (0.56, 0.75)	<0.001
	C3	0.71 (0.65, 0.77)		0.81 (0.75, 0.88)		0.64 (0.56, 0.74)		0.59 (0.49, 0.69)		0.51 (0.44, 0.60)	
	C4	0.60 (0.53, 0.68)		0.89 (0.82, 0.98)		0.56 (0.47, 0.66)		0.64 (0.53, 0.78)		0.47 (0.39, 0.56)	
ELIH	C1	1.00		1.00		1.00		1.00		1.00	
	C2	0.87 (0.80, 0.94)	<0.001	0.98 (0.91, 1.05)	0.012	0.82 (0.73, 0.93)	<0.001	0.92 (0.79, 1.08)	0.018	0.72 (0.62, 0.82)	<0.001
	C3	0.76 (0.70, 0.83)		0.89 (0.83, 0.96)		0.65 (0.56, 0.75)		0.92 (0.78, 1.08)		0.59 (0.50, 0.69)	
	C4	0.73 (0.67, 0.80)		0.93 (0.86, 1.01)		0.63 (0.54, 0.73)		0.79 (0.67, 0.95)		0.58 (0.49, 0.68)	
ELIR	C1	1.00		1.00		1.00		1.00		1.00	
	C2	0.86 (0.79, 0.94)	<0.001	0.97 (0.90, 1.04)	0.590	0.93 (0.82, 1.05)	<0.001	0.96 (0.82, 1.13)	0.588	0.80 (0.69, 0.92)	<0.001
	C3	0.83 (0.76, 0.90)		0.97 (0.90, 1.05)		0.72 (0.63, 0.83)		0.96 (0.81, 1.13)		0.67 (0.57, 0.78)	
	C4	0.83 (0.76, 0.91)		1.02 (0.95, 1.10)		0.72 (0.62, 0.84)		1.06 (0.89, 1.25)		0.73 (0.62, 0.85)	
HB	C1	1.00		1.00		1.00		1.00		1.00	
	C2	0.75 (0.67, 0.84)	<0.001	0.87 (0.79, 0.95)	<0.001	0.91 (0.76, 1.09)	0.046	0.74 (0.60, 0.92)	0.037	0.80 (0.66, 0.98)	<0.001
	C3	0.66 (0.59, 0.74)		0.82 (0.75, 0.90)		0.82 (0.69, 0.98)		0.78 (0.63, 0.96)		0.69 (0.57, 0.84)	
	C4	0.59 (0.53, 0.67)		0.77 (0.69, 0.85)		0.85 (0.70, 1.03)		0.71 (0.56, 0.89)		0.68 (0.55, 0.83)	
HLI	C1	1.00		1.00		1.00		1.00		1.00	
	C2	0.89 (0.82, 0.97)	<0.001	0.96 (0.90, 1.03)	<0.001	0.83 (0.73, 0.94)	<0.001	0.86 (0.74, 1.01)	0.011	0.72 (0.62, 0.84)	<0.001
	C3	0.80 (0.74, 0.87)		0.89 (0.83, 0.96)		0.78 (0.68, 0.89)		0.83 (0.71, 0.98)		0.72 (0.62, 0.84)	
	C4	0.70 (0.64, 0.77)		0.85 (0.79, 0.92)		0.79 (0.69, 0.92)		0.81 (0.68, 0.96)		0.63 (0.53, 0.74)	
HLI_{WHR}	C1	1.00		1.00		1.00		1.00		1.00	
	C2	0.84 (0.77, 0.91)	<0.001	0.93 (0.87, 1.00)	<0.001	0.91 (0.80, 1.04)	<0.001	0.90 (0.77, 1.06)	0.065	0.72 (0.62, 0.84)	<0.001
	C3	0.72 (0.66, 0.79)		0.87 (0.81, 0.94)		0.87 (0.76, 1.00)		0.92 (0.78, 1.08)		0.80 (0.69, 0.93)	
	C4	0.71 (0.65, 0.78)		0.87 (0.80, 0.94)		0.72 (0.62, 0.85)		0.83 (0.70, 1.00)		0.63 (0.53, 0.75)	
HLI_{WST}	C1	1.00		1.00		1.00		1.00		1.00	
	C2	0.79 (0.72, 0.86)	<0.001	0.87 (0.81, 0.93)	<0.001	0.93 (0.82, 1.06)	0.003	0.78 (0.67, 0.91)	0.015	0.74 (0.64, 0.85)	<0.001
	C3	0.79 (0.72, 0.85)		0.87 (0.81, 0.93)		0.84 (0.73, 0.97)		0.85 (0.73, 1.00)		0.75 (0.65, 0.88)	
	C4	0.69 (0.63, 0.76)		0.85 (0.79, 0.92)		0.82 (0.70, 0.95)		0.78 (0.65, 0.93)		0.68 (0.57, 0.80)	
HLS	C1	1.00		1.00		1.00		1.00		1.00	
	C2	0.76 (0.70, 0.82)	<0.001	0.89 (0.83, 0.96)	<0.001	0.77 (0.67, 0.87)	0.001	0.94 (0.81, 1.10)	0.007	0.84 (0.73, 0.96)	<0.001
	C3	0.66 (0.61, 0.72)		0.84 (0.78, 0.90)		0.77 (0.67, 0.88)		0.89 (0.76, 1.05)		0.63 (0.54, 0.74)	

Lifestyle score	Category	Lifestyle-related cancer incidence		Other cancer incidence		MI incidence		Stroke incidence		HF incidence	
		HR (95% CI)	P_{trend}	HR (95% CI)	P_{trend}	HR (95% CI)	P_{trend}	HR (95% CI)	P_{trend}	HR (95% CI)	P_{trend}
	C4	0.55 (0.49, 0.62)		0.76 (0.69, 0.84)		0.77 (0.64, 0.92)		0.71 (0.57, 0.90)		0.54 (0.43, 0.68)	
LIS	C1	1.00	<0.001	1.00	<0.001	1.00	0.014	1.00	<0.001	1.00	<0.001
	C2	1.00 (0.91, 1.09)		1.02 (0.94, 1.10)		1.00 (0.87, 1.16)		1.01 (0.85, 1.19)		0.89 (0.76, 1.04)	
	C3	0.77 (0.71, 0.83)		0.88 (0.82, 0.94)		0.91 (0.80, 1.03)		0.83 (0.72, 0.96)		0.65 (0.57, 0.75)	
	C4	0.68 (0.62, 0.75)		0.84 (0.78, 0.91)		0.85 (0.73, 0.98)		0.76 (0.64, 0.90)		0.57 (0.48, 0.67)	
LRLS	C1	1.00	<0.001	1.00	<0.001	1.00	0.001	1.00	0.156	1.00	<0.001
	C2	0.91 (0.84, 0.98)		0.94 (0.87, 1.01)		0.95 (0.84, 1.09)		0.94 (0.80, 1.09)		0.85 (0.74, 0.98)	
	C3	0.78 (0.72, 0.85)		0.87 (0.81, 0.94)		0.85 (0.74, 0.98)		0.92 (0.78, 1.08)		0.70 (0.60, 0.81)	
	C4	0.69 (0.61, 0.79)		0.87 (0.79, 0.96)		0.77 (0.64, 0.92)		0.86 (0.69, 1.06)		0.56 (0.45, 0.70)	
MEDLIFE	C1	1.00	<0.001	1.00	0.006	1.00	0.025	1.00	0.361	1.00	0.107
	C2	0.95 (0.87, 1.04)		0.98 (0.91, 1.06)		0.94 (0.82, 1.08)		0.94 (0.80, 1.12)		0.87 (0.74, 1.02)	
	C3	0.90 (0.82, 0.98)		1.00 (0.93, 1.08)		0.95 (0.82, 1.09)		1.02 (0.86, 1.20)		0.88 (0.75, 1.03)	
	C4	0.82 (0.76, 0.89)		0.90 (0.84, 0.96)		0.85 (0.75, 0.97)		0.91 (0.78, 1.07)		0.88 (0.76, 1.02)	
WCRF/AICR	C1	1.00	<0.001	1.00	0.008	1.00	<0.001	1.00	<0.001	1.00	<0.001
	C2	0.86 (0.78, 0.94)		0.97 (0.90, 1.04)		0.88 (0.77, 1.01)		0.91 (0.77, 1.07)		0.82 (0.70, 0.95)	
	C3	0.80 (0.74, 0.87)		0.94 (0.88, 1.01)		0.80 (0.70, 0.91)		0.82 (0.70, 0.96)		0.78 (0.68, 0.91)	
	C4	0.71 (0.65, 0.78)		0.90 (0.83, 0.98)		0.78 (0.67, 0.91)		0.75 (0.62, 0.89)		0.64 (0.54, 0.77)	

Cox proportional hazards regression adjusted for age and sex, ethnicity, region, index of multiple deprivation, household income, education, middle degree, drug use, history of cancer screening, family history of diabetes, cancer, or CVD. The variable used for stratification was not adjusted for. Abbreviations: NCD, non-communicable disease; ACS, the American Cancer Society guidelines score; CDRI, chronic disease risk index; ELIH, empirical lifestyle pattern score for hyperinsulinemia; ELIR, empirical lifestyle pattern score for insulin resistance; HB, Health behaviors; HLI, healthy lifestyle index; HLS, healthy lifestyle score; LIS, lifestyle inflammation score; LRLS, low-risk lifestyle score; MEDLIFE, the Mediterranean lifestyle; WCRF/AICR score, World Cancer Research Fund and the American Institute for Cancer Research score.

Supplementary Table 6. Associations of 13 lifestyle scores with primary NCD-related outcomes mortality

Lifestyle score	Category	All-cause mortality		NCD mortality		Cancer mortality		CVD mortality	
		**HR (95% CI)**	P_{trend}	**HR (95% CI)**	P_{trend}	**HR (95% CI)**	P_{trend}	**HR (95% CI)**	P_{trend}
Cases/person-years	3,056/807,956			2,250/ 807,956		1,687/807,956		561/807,956	
ACS	C1	1.00	<0.001	1.00	<0.001	1.00	<0.001	1.00	0.018
	C2	0.93 (0.85, 1.02)		0.97 (0.87, 1.08)		0.97 (0.86, 1.10)		0.98 (0.79, 1.21)	
	C3	0.85 (0.77, 0.93)		0.89 (0.80, 0.99)		0.88 (0.78, 1.00)		0.92 (0.73, 1.14)	
	C4	0.77 (0.68, 0.86)		0.73 (0.63, 0.84)		0.74 (0.63, 0.87)		0.69 (0.52, 0.92)	
CDRI	C1	1.00	<0.001	1.00	<0.001	1.00	<0.001	1.00	<0.001
	C2	0.69 (0.62, 0.76)		0.69 (0.61, 0.78)		0.73 (0.64, 0.84)		0.59 (0.47, 0.74)	
	C3	0.59 (0.53, 0.65)		0.56 (0.50, 0.64)		0.58 (0.51, 0.67)		0.53 (0.42, 0.66)	
	C4	0.52 (0.46, 0.59)		0.51 (0.44, 0.59)		0.54 (0.46, 0.64)		0.44 (0.34, 0.59)	
ELIH	C1	1.00	<0.001	1.00	<0.001	1.00	<0.001	1.00	<0.001
	C2	0.86 (0.78, 0.94)		0.84 (0.76, 0.94)		0.85 (0.75, 0.97)		0.81 (0.66, 1.00)	
	C3	0.76 (0.69, 0.84)		0.73 (0.65, 0.82)		0.76 (0.66, 0.87)		0.66 (0.52, 0.83)	
	C4	0.79 (0.71, 0.88)		0.70 (0.62, 0.80)		0.75 (0.65, 0.86)		0.58 (0.45, 0.75)	
ELIR	C1	1.00	0.434	1.00	0.019	1.00	0.088	1.00	0.073
	C2	0.88 (0.79, 0.97)		0.83 (0.74, 0.92)		0.85 (0.74, 0.97)		0.77 (0.61, 0.96)	
	C3	0.86 (0.78, 0.95)		0.76 (0.67, 0.86)		0.77 (0.67, 0.88)		0.74 (0.59, 0.94)	
	C4	0.97 (0.88, 1.07)		0.90 (0.80, 1.01)		0.92 (0.80, 1.05)		0.83 (0.66, 1.05)	
HB	C1	1.00	<0.001	1.00	<0.001	1.00	<0.001	1.00	0.172
	C2	0.72 (0.64, 0.81)		0.71 (0.62, 0.82)		0.70 (0.60, 0.83)		0.73 (0.55, 0.98)	
	C3	0.63 (0.56, 0.71)		0.64 (0.56, 0.73)		0.62 (0.53, 0.73)		0.70 (0.53, 0.93)	
	C4	0.58 (0.51, 0.67)		0.58 (0.50, 0.68)		0.53 (0.45, 0.64)		0.75 (0.55, 1.02)	
HLI	C1	1.00	<0.001	1.00	<0.001	1.00	<0.001	1.00	0.076
	C2	0.94 (0.86, 1.03)		0.94 (0.85, 1.05)		0.99 (0.87, 1.12)		0.82 (0.66, 1.02)	
	C3	0.83 (0.75, 0.92)		0.79 (0.70, 0.89)		0.77 (0.67, 0.88)		0.87 (0.69, 1.08)	
	C4	0.79 (0.71, 0.88)		0.73 (0.65, 0.83)		0.72 (0.62, 0.83)		0.79 (0.62, 1.01)	
HLI_{WHR}	C1	1.00	<0.001	1.00	<0.001	1.00	<0.001	1.00	0.087
	C2	0.87 (0.79, 0.96)		0.89 (0.79, 0.99)		0.87 (0.77, 0.99)		0.94 (0.75, 1.16)	
	C3	0.79 (0.72, 0.88)		0.78 (0.70, 0.88)		0.74 (0.65, 0.85)		0.93 (0.74, 1.17)	
	C4	0.75 (0.67, 0.83)		0.74 (0.65, 0.83)		0.72 (0.63, 0.83)		0.78 (0.60, 1.01)	
HLI_{WST}	C1	1.00	<0.001	1.00	<0.001	1.00	<0.001	1.00	0.074
	C2	0.85 (0.77, 0.93)		0.85 (0.76, 0.94)		0.85 (0.75, 0.96)		0.85 (0.68, 1.05)	
	C3	0.83 (0.75, 0.91)		0.79 (0.70, 0.88)		0.76 (0.66, 0.87)		0.88 (0.70, 1.10)	
	C4	0.75 (0.67, 0.84)		0.75 (0.66, 0.85)		0.74 (0.64, 0.85)		0.77 (0.60, 1.00)	
HLS	C1	1.00	<0.001	1.00	<0.001	1.00	<0.001	1.00	0.004
	C2	0.79 (0.72, 0.86)		0.79 (0.71, 0.87)		0.77 (0.68, 0.86)		0.86 (0.70, 1.07)	
	C3	0.65 (0.59, 0.71)		0.66 (0.59, 0.73)		0.62 (0.54, 0.70)		0.80 (0.64, 1.00)	

Lifestyle score	Category	All-cause mortality		NCD mortality		Cancer mortality		CVD mortality	
		HR (95% CI)	P_{trend}	HR (95% CI)	P_{trend}	HR (95% CI)	P_{trend}	HR (95% CI)	P_{trend}
	C4	0.62 (0.54, 0.71)		0.54 (0.45, 0.63)		0.52 (0.43, 0.62)		0.62 (0.44, 0.87)	
LIS	C1	1.00	<0.001	1.00	<0.001	1.00	<0.001	1.00	0.009
	C2	1.09 (0.98, 1.20)		1.05 (0.94, 1.18)		1.01 (0.89, 1.16)		1.17 (0.93, 1.48)	
	C3	0.80 (0.73, 0.87)		0.82 (0.74, 0.91)		0.80 (0.71, 0.90)		0.89 (0.72, 1.10)	
	C4	0.73 (0.65, 0.81)		0.64 (0.57, 0.73)		0.62 (0.54, 0.72)		0.72 (0.56, 0.93)	
LRLS	C1	1.00	<0.001	1.00	<0.001	1.00	<0.001	1.00	0.011
	C2	0.93 (0.85, 1.02)		0.92 (0.83, 1.02)		0.96 (0.85, 1.09)		0.79 (0.63, 0.98)	
	C3	0.81 (0.74, 0.90)		0.78 (0.70, 0.88)		0.76 (0.66, 0.87)		0.85 (0.68, 1.06)	
	C4	0.69 (0.60, 0.80)		0.66 (0.56, 0.78)		0.68 (0.56, 0.82)		0.62 (0.45, 0.85)	
MEDLIFE	C1	1.00	<0.001	1.00	<0.001	1.00	<0.001	1.00	0.710
	C2	0.90 (0.81, 0.99)		0.92 (0.82, 1.03)		0.90 (0.79, 1.03)		0.94 (0.74, 1.20)	
	C3	0.85 (0.76, 0.94)		0.84 (0.74, 0.94)		0.81 (0.71, 0.93)		0.91 (0.72, 1.16)	
	C4	0.79 (0.72, 0.87)		0.80 (0.71, 0.89)		0.74 (0.65, 0.84)		0.97 (0.78, 1.20)	
WCRF/AICR	C1	1.00	<0.001	1.00	<0.001	1.00	<0.001	1.00	0.007
	C2	0.89 (0.81, 0.98)		0.92 (0.82, 1.04)		0.92 (0.81, 1.05)		0.93 (0.74, 1.17)	
	C3	0.78 (0.71, 0.86)		0.78 (0.70, 0.88)		0.79 (0.70, 0.90)		0.76 (0.60, 0.95)	
	C4	0.72 (0.64, 0.80)		0.73 (0.65, 0.83)		0.73 (0.63, 0.84)		0.76 (0.59, 0.98)	

Cox proportional hazards regression adjusted for age and sex, ethnicity, region, index of multiple deprivation, household income, education, middle degree, drug use, history of cancer screening, family history of diabetes, cancer, or CVD. The variable used for stratification was not adjusted for. Abbreviations: NCD, non-communicable disease; ACS, the American Cancer Society guidelines score; CDRI, chronic disease risk index; ELIH, empirical lifestyle pattern score for hyperinsulinemia; ELIR, empirical lifestyle pattern score for insulin resistance; HB, Health behaviors; HLI, healthy lifestyle index; HLS, healthy lifestyle score; LIS, lifestyle inflammation score; LRLS, low-risk lifestyle score; MEDLIFE, the Mediterranean lifestyle; WCRF/AICR score, World Cancer Research Fund and the American Institute for Cancer Research score.

Supplementary Table 7. Associations of 13 lifestyle scores with secondary NCD-related outcomes mortality

Lifestyle score	Category	Lifestyle-related cancer mortality		Other cancer mortality	
		HR (95% CI)	P_{trend}	HR (95% CI)	P_{trend}
Cases/ person-years		1,044/807,956		643/807,956	
ACS	C1	1.00	<0.001	1.00	0.354
	C2	0.92 (0.79, 1.08)		1.06 (0.87, 1.31)	
	C3	0.78 (0.66, 0.92)		1.08 (0.87, 1.32)	
	C4	0.70 (0.57, 0.85)		0.83 (0.64, 1.08)	
CDRI	C1	1.00	<0.001	1.00	0.399
	C2	0.64 (0.54, 0.75)		0.96 (0.76, 1.23)	
	C3	0.49 (0.42, 0.58)		0.81 (0.64, 1.03)	
	C4	0.35 (0.28, 0.45)		0.97 (0.74, 1.27)	
ELIH	C1	1.00	<0.001	1.00	0.224
	C2	0.75 (0.64, 0.88)		1.05 (0.85, 1.29)	
	C3	0.65 (0.55, 0.77)		0.97 (0.78, 1.21)	
	C4	0.68 (0.57, 0.81)		0.88 (0.69, 1.11)	
ELIR	C1	1.00	0.009	1.00	0.570
	C2	0.77 (0.65, 0.91)		1.01 (0.81, 1.25)	
	C3	0.65 (0.55, 0.78)		1.00 (0.80, 1.24)	
	C4	0.84 (0.71, 0.99)		1.07 (0.86, 1.34)	
HB	C1	1.00	<0.001	1.00	0.021
	C2	0.72 (0.58, 0.88)		0.68 (0.52, 0.89)	
	C3	0.56 (0.46, 0.69)		0.73 (0.56, 0.95)	
	C4	0.48 (0.39, 0.61)		0.62 (0.47, 0.83)	
HLI	C1	1.00	<0.001	1.00	0.485
	C2	0.92 (0.79, 1.08)		1.11 (0.90, 1.37)	
	C3	0.67 (0.57, 0.80)		0.96 (0.77, 1.20)	
	C4	0.60 (0.50, 0.72)		0.96 (0.76, 1.21)	
HLI_{WHR}	C1	1.00	<0.001	1.00	0.481
	C2	0.79 (0.67, 0.94)		1.00 (0.81, 1.23)	
	C3	0.65 (0.54, 0.77)		0.91 (0.73, 1.14)	
	C4	0.61 (0.51, 0.74)		0.95 (0.75, 1.20)	
HLI_{WST}	C1	1.00	<0.001	1.00	0.677
	C2	0.79 (0.67, 0.93)		0.95 (0.77, 1.16)	
	C3	0.69 (0.59, 0.82)		0.87 (0.70, 1.08)	
	C4	0.61 (0.51, 0.74)		1.00 (0.79, 1.26)	
HLS	C1	1.00	<0.001	1.00	0.070
	C2	0.66 (0.57, 0.76)		1.02 (0.83, 1.25)	
	C3	0.51 (0.43, 0.60)		0.88 (0.71, 1.09)	

Lifestyle score	Category	Lifestyle-related cancer mortality		Other cancer mortality	
		HR (95% CI)	P_{trend}	HR (95% CI)	P_{trend}
	C4	0.39 (0.30, 0.50)		0.82 (0.61, 1.10)	
LIS	C1	1.00	<0.001	1.00	0.041
	C2	1.03 (0.87, 1.21)		0.98 (0.78, 1.23)	
	C3	0.71 (0.60, 0.82)		0.96 (0.80, 1.17)	
	C4	0.55 (0.45, 0.66)		0.76 (0.60, 0.96)	
LRLS	C1	1.00	<0.001	1.00	0.166
	C2	0.96 (0.82, 1.11)		0.98 (0.80, 1.20)	
	C3	0.71 (0.60, 0.85)		0.84 (0.68, 1.05)	
	C4	0.54 (0.41, 0.70)		0.91 (0.69, 1.19)	
MEDLIFE	C1	1.00	<0.001	1.00	0.216
	C2	0.84 (0.71, 1.00)		1.01 (0.81, 1.26)	
	C3	0.75 (0.63, 0.90)		0.91 (0.73, 1.14)	
	C4	0.66 (0.56, 0.77)		0.90 (0.73, 1.11)	
WCRF/AICR	C1	1.00	<0.001	1.00	0.109
	C2	0.78 (0.66, 0.92)		1.23 (0.99, 1.54)	
	C3	0.67 (0.57, 0.79)		1.06 (0.85, 1.32)	
	C4	0.62 (0.52, 0.75)		0.97 (0.76, 1.24)	

Cox proportional hazards regression adjusted for age and sex, ethnicity, region, index of multiple deprivation, household income, education, middle degree, drug use, history of cancer screening, family history of diabetes, cancer, or CVD. The variable used for stratification was not adjusted for. Abbreviations: NCD, non-communicable disease; ACS, the American Cancer Society guidelines score; CDRI, chronic disease risk index; ELIH, empirical lifestyle pattern score for hyperinsulinemia; ELIR, empirical lifestyle pattern score for insulin resistance; HB, Health behaviors; HLI, healthy lifestyle index; HLS, healthy lifestyle score; LIS, lifestyle inflammation score; LRLS, low-risk lifestyle score; MEDLIFE, the Mediterranean lifestyle; WCRF/AICR score, World Cancer Research Fund and the American Institute for Cancer Research score.

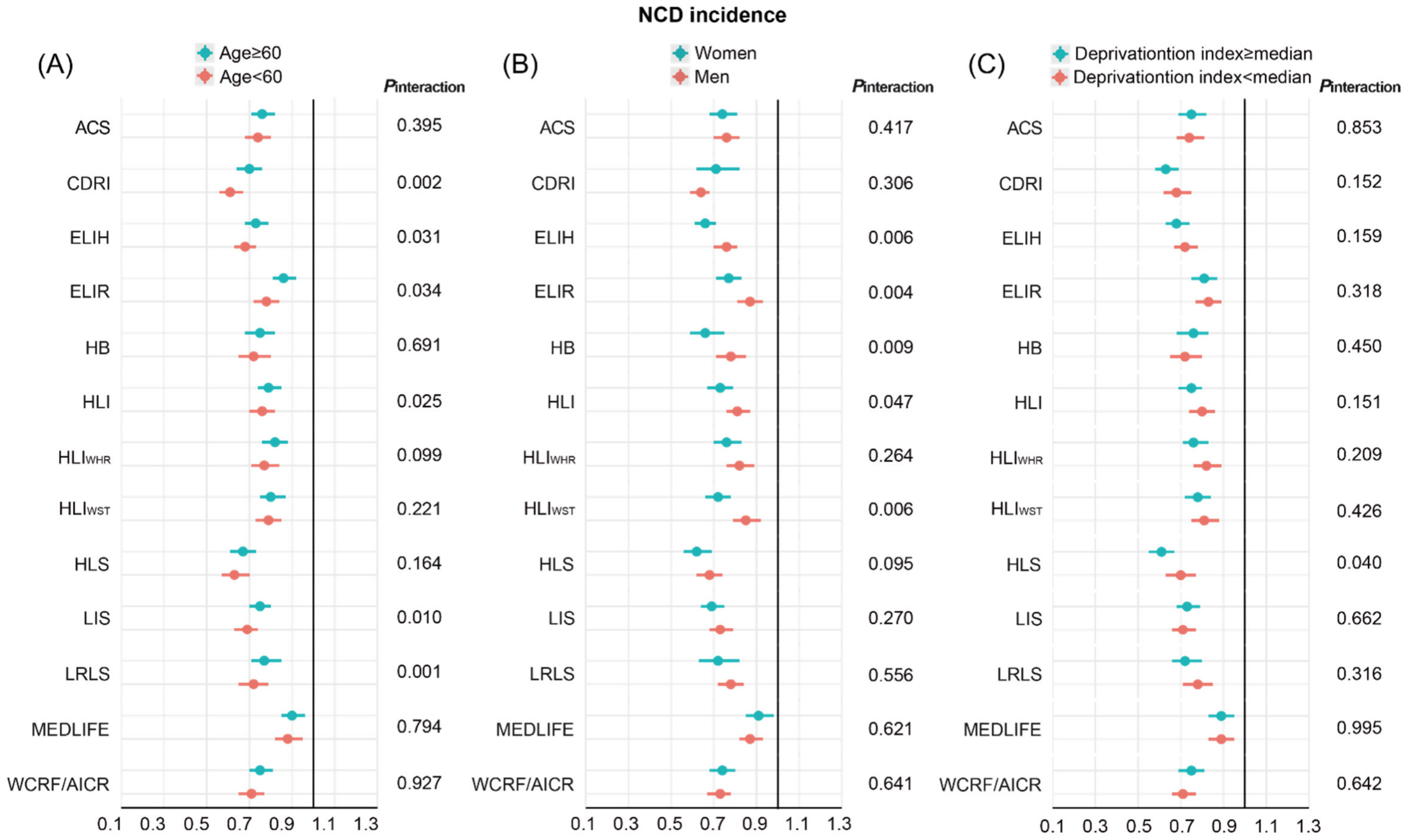

Supplementary Figure 2. Fully-adjusted HR and 95% CI for major NCD incidence in subgroups defined by (A) age, (B) sex, and (C) deprivation index, comparing the highest to the lowest category of each lifestyle score

Cox proportional hazards regression adjusted for age and sex, ethnicity, region, index of multiple deprivation, household income, education, middle degree, drug use, history of cancer screening, family history of diabetes, cancer, or CVD. The variable used for stratification was not adjusted for. Abbreviations: NCD, non-communicable disease; ACS, the American Cancer Society guidelines score; CDRI, chronic disease risk index; ELIH, empirical lifestyle pattern score for hyperinsulinemia; ELIR, empirical lifestyle pattern score for insulin resistance; HB, Health behaviors; HLI, healthy lifestyle index; HLS, healthy lifestyle score; LIS, lifestyle inflammation score; LRLS, low-risk lifestyle score; MEDLIFE, the Mediterranean lifestyle; WCRF/AICR score, World Cancer Research Fund and the American Institute for Cancer Research score.

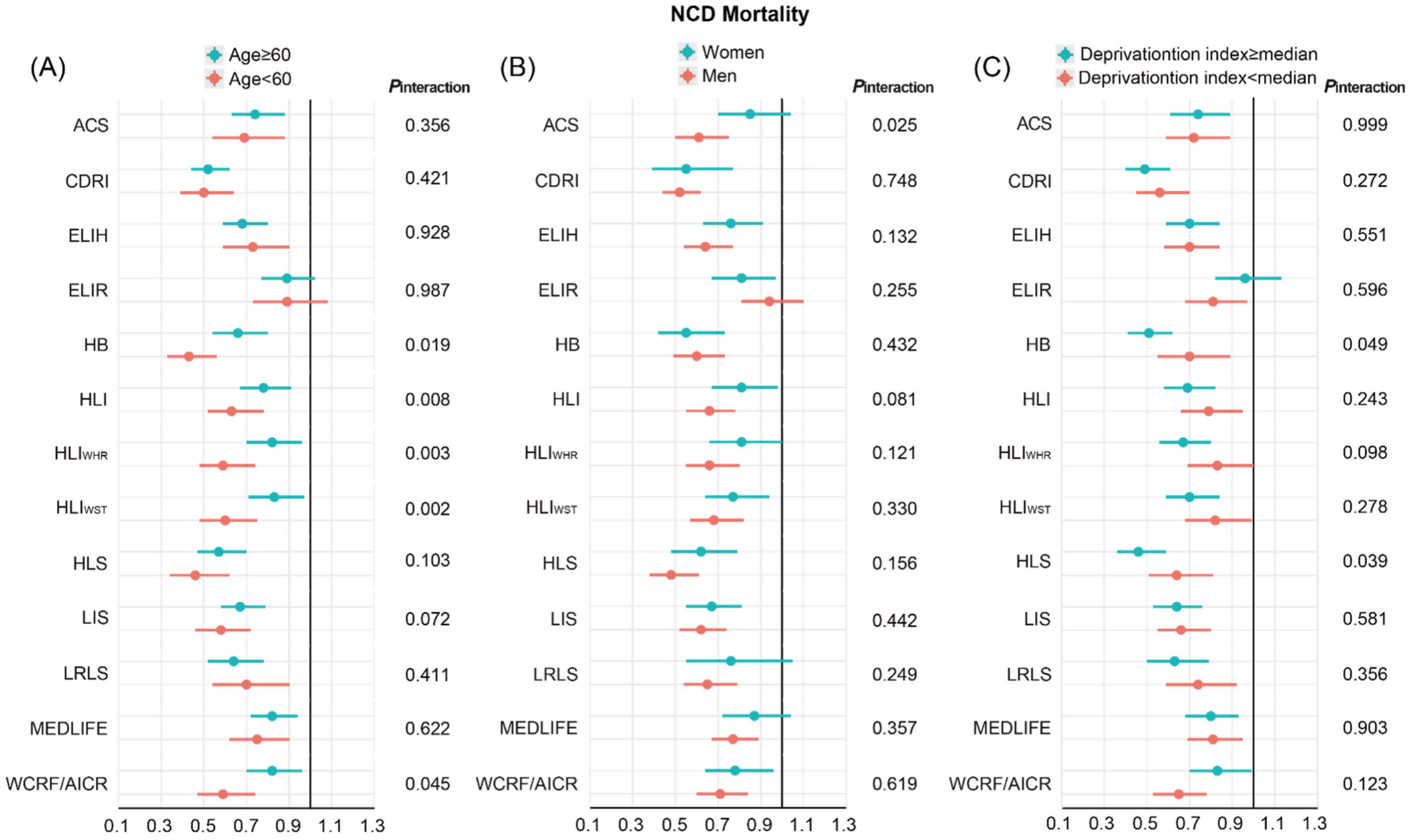

Supplementary Figure 3. Fully-adjusted hazard ratios and 95% confidence intervals for major NCD mortality in subgroups defined by (A) age, (B) sex, and (C) deprivation index, comparing the highest to the lowest category of each lifestyle score

Cox proportional hazards regression adjusted for age and sex, ethnicity, region, index of multiple deprivation, household income, education, middle degree, drug use, history of cancer screening, family history of diabetes, cancer, or CVD. The variable used for stratification was not adjusted for. Abbreviations: NCD, non-communicable disease; ACS, the American Cancer Society guidelines score; CDRI, chronic disease risk index; ELIH, empirical lifestyle pattern score for hyperinsulinemia; ELIR, empirical lifestyle pattern score for insulin resistance; HB, Health behaviors; HLI, healthy lifestyle index; HLS, healthy lifestyle score; LIS, lifestyle inflammation score; LRLS, low-risk lifestyle score; MEDLIFE, the Mediterranean lifestyle; WCRF/AICR score, World Cancer Research Fund and the American Institute for Cancer Research score.

Supplementary Table 8. Associations of 13 lifestyle scores with major NCD incidence in subgroups

Subgroup		Age		Sex		Deprivation index	
**Lifestyle score**	**Category**	**<60**	**≥60**	**Women**	**Men**	**<Median**	**≥Median**
ACS	C1	1.00	1.00	1.00	1.00	1.00	1.00
	C2	0.90 (0.84, 0.97)	0.91 (0.85, 0.97)	0.90 (0.83, 0.97)	0.91 (0.86, 0.96)	0.91 (0.85, 0.97)	0.89 (0.84, 0.95)
	C3	0.83 (0.77, 0.89)	0.85 (0.79, 0.90)	0.84 (0.78, 0.90)	0.84 (0.79, 0.89)	0.82 (0.77, 0.88)	0.84 (0.79, 0.90)
	C4	0.74 (0.68, 0.80)	0.76 (0.71, 0.82)	0.74 (0.68, 0.81)	0.76 (0.70, 0.82)	0.74 (0.68, 0.81)	0.75 (0.69, 0.82)
CDRI	C1	1.00	1.00	1.00	1.00	1.00	1.00
	C2	0.70 (0.65, 0.76)	0.83 (0.77, 0.89)	0.78 (0.72, 0.85)	0.77 (0.72, 0.82)	0.81 (0.74, 0.88)	0.74 (0.69, 0.79)
	C3	0.63 (0.58, 0.68)	0.70 (0.65, 0.75)	0.70 (0.65, 0.77)	0.64 (0.60, 0.68)	0.71 (0.66, 0.77)	0.63 (0.58, 0.67)
	C4	0.61 (0.56, 0.67)	0.70 (0.64, 0.76)	0.71 (0.62, 0.82)	0.64 (0.59, 0.68)	0.68 (0.62, 0.75)	0.63 (0.58, 0.69)
ELIH	C1	1.00	1.00	1.00	1.00	1.00	1.00
	C2	0.78 (0.73, 0.84)	0.83 (0.78, 0.88)	0.80 (0.74, 0.86)	0.81 (0.77, 0.86)	0.80 (0.75, 0.86)	0.81 (0.76, 0.87)
	C3	0.68 (0.63, 0.73)	0.73 (0.68, 0.78)	0.70 (0.64, 0.75)	0.70 (0.66, 0.75)	0.69 (0.64, 0.74)	0.70 (0.65, 0.75)
	C4	0.68 (0.63, 0.73)	0.73 (0.68, 0.79)	0.66 (0.61, 0.71)	0.76 (0.70, 0.81)	0.72 (0.67, 0.78)	0.68 (0.63, 0.74)
ELIR	C1	1.00	1.00	1.00	1.00	1.00	1.00
	C2	0.82 (0.76, 0.88)	0.89 (0.83, 0.95)	0.83 (0.77, 0.90)	0.86 (0.81, 0.92)	0.86 (0.80, 0.92)	0.84 (0.79, 0.90)
	C3	0.74 (0.68, 0.80)	0.84 (0.78, 0.90)	0.76 (0.70, 0.82)	0.81 (0.76, 0.86)	0.79 (0.74, 0.85)	0.79 (0.73, 0.84)
	C4	0.78 (0.72, 0.84)	0.86 (0.81, 0.92)	0.77 (0.71, 0.83)	0.87 (0.81, 0.93)	0.83 (0.77, 0.89)	0.81 (0.75, 0.87)
HB	C1	1.00	1.00	1.00	1.00	1.00	1.00
	C2	0.86 (0.78, 0.94)	0.85 (0.78, 0.93)	0.80 (0.70, 0.90)	0.87 (0.81, 0.94)	0.84 (0.76, 0.92)	0.88 (0.80, 0.96)
	C3	0.79 (0.72, 0.86)	0.80 (0.73, 0.87)	0.74 (0.65, 0.83)	0.81 (0.75, 0.87)	0.77 (0.70, 0.85)	0.81 (0.74, 0.89)
	C4	0.72 (0.65, 0.80)	0.75 (0.68, 0.82)	0.66 (0.59, 0.75)	0.78 (0.71, 0.85)	0.72 (0.65, 0.80)	0.76 (0.68, 0.83)
HLI	C1	1.00	1.00	1.00	1.00	1.00	1.00
	C2	0.88 (0.82, 0.95)	0.90 (0.84, 0.96)	0.86 (0.79, 0.93)	0.91 (0.86, 0.97)	0.90 (0.84, 0.97)	0.89 (0.83, 0.95)
	C3	0.81 (0.75, 0.87)	0.86 (0.81, 0.92)	0.82 (0.76, 0.89)	0.84 (0.79, 0.90)	0.83 (0.77, 0.89)	0.85 (0.79, 0.91)
	C4	0.76 (0.70, 0.82)	0.79 (0.74, 0.85)	0.73 (0.67, 0.79)	0.81 (0.76, 0.87)	0.80 (0.74, 0.86)	0.75 (0.69, 0.80)
HLI_{WHR}	C1	1.00	1.00	1.00	1.00	1.00	1.00
	C2	0.91 (0.84, 0.98)	0.90 (0.84, 0.95)	0.91 (0.82, 1.00)	0.89 (0.84, 0.94)	0.91 (0.85, 0.98)	0.90 (0.84, 0.96)
	C3	0.83 (0.77, 0.90)	0.87 (0.81, 0.93)	0.81 (0.74, 0.89)	0.88 (0.82, 0.93)	0.87 (0.81, 0.93)	0.84 (0.78, 0.90)
	C4	0.77 (0.71, 0.84)	0.82 (0.76, 0.88)	0.76 (0.70, 0.83)	0.82 (0.76, 0.89)	0.82 (0.76, 0.89)	0.76 (0.71, 0.83)
HLI_{WST}	C1	1.00	1.00	1.00	1.00	1.00	1.00
	C2	0.87 (0.81, 0.94)	0.86 (0.80, 0.91)	0.80 (0.74, 0.87)	0.89 (0.84, 0.94)	0.87 (0.81, 0.93)	0.86 (0.80, 0.92)
	C3	0.85 (0.79, 0.91)	0.87 (0.82, 0.93)	0.80 (0.74, 0.86)	0.89 (0.84, 0.95)	0.88 (0.82, 0.95)	0.84 (0.78, 0.90)
	C4	0.79 (0.73, 0.85)	0.80 (0.75, 0.87)	0.72 (0.66, 0.78)	0.85 (0.79, 0.92)	0.81 (0.75, 0.88)	0.78 (0.72, 0.84)
HLS	C1	1.00	1.00	1.00	1.00	1.00	1.00
	C2	0.86 (0.81, 0.92)	0.83 (0.78, 0.88)	0.82 (0.76, 0.89)	0.85 (0.81, 0.91)	0.87 (0.81, 0.93)	0.81 (0.76, 0.87)
	C3	0.75 (0.70, 0.81)	0.78 (0.74, 0.84)	0.78 (0.72, 0.84)	0.76 (0.71, 0.81)	0.78 (0.73, 0.84)	0.75 (0.70, 0.80)
	C4	0.63 (0.57, 0.70)	0.67 (0.61, 0.73)	0.62 (0.56, 0.69)	0.68 (0.62, 0.74)	0.70 (0.63, 0.77)	0.61 (0.55, 0.67)

Subgroup		Age		Sex		Deprivation index	
**Lifestyle score**	**Category**	**<60**	**≥60**	**Women**	**Men**	**<Median**	**≥Median**
LIS	C1	1.00	1.00	1.00	1.00	1.00	1.00
	C2	1.00 (0.93, 1.07)	0.99 (0.93, 1.07)	0.95 (0.88, 1.03)	1.02 (0.96, 1.09)	1.02 (0.95, 1.10)	0.97 (0.91, 1.04)
	C3	0.71 (0.67, 0.77)	0.83 (0.78, 0.88)	0.74 (0.69, 0.79)	0.80 (0.75, 0.85)	0.81 (0.76, 0.86)	0.74 (0.69, 0.79)
	C4	0.69 (0.63, 0.74)	0.75 (0.70, 0.80)	0.69 (0.64, 0.75)	0.73 (0.68, 0.79)	0.71 (0.66, 0.77)	0.73 (0.68, 0.79)
LRLS	C1	1.00	1.00	1.00	1.00	1.00	1.00
	C2	0.87 (0.81, 0.94)	0.94 (0.88, 0.99)	0.86 (0.80, 0.92)	0.95 (0.89, 1.02)	0.97 (0.90, 1.03)	0.86 (0.81, 0.92)
	C3	0.80 (0.74, 0.86)	0.83 (0.78, 0.89)	0.74 (0.69, 0.80)	0.88 (0.82, 0.94)	0.82 (0.77, 0.89)	0.81 (0.75, 0.87)
	C4	0.72 (0.65, 0.79)	0.77 (0.71, 0.85)	0.72 (0.63, 0.82)	0.78 (0.72, 0.84)	0.78 (0.71, 0.85)	0.72 (0.66, 0.80)
MEDLIFE	C1	1.00	1.00	1.00	1.00	1.00	1.00
	C2	0.96 (0.89, 1.04)	0.99 (0.92, 1.06)	0.97 (0.89, 1.06)	0.98 (0.92, 1.04)	0.95 (0.88, 1.02)	1.01 (0.94, 1.08)
	C3	0.95 (0.88, 1.02)	0.98 (0.92, 1.05)	0.97 (0.89, 1.05)	0.97 (0.91, 1.03)	0.98 (0.91, 1.06)	0.95 (0.88, 1.02)
	C4	0.88 (0.82, 0.95)	0.90 (0.85, 0.96)	0.91 (0.85, 0.98)	0.87 (0.82, 0.93)	0.89 (0.83, 0.95)	0.89 (0.83, 0.95)
WCRF/AICR	C1	1.00	1.00	1.00	1.00	1.00	1.00
	C2	0.83 (0.78, 0.90)	0.90 (0.84, 0.96)	0.87 (0.80, 0.95)	0.86 (0.81, 0.92)	0.87 (0.81, 0.93)	0.85 (0.79, 0.92)
	C3	0.78 (0.73, 0.84)	0.85 (0.79, 0.90)	0.83 (0.77, 0.90)	0.80 (0.75, 0.85)	0.81 (0.76, 0.87)	0.81 (0.75, 0.86)
	C4	0.71 (0.65, 0.77)	0.75 (0.70, 0.81)	0.74 (0.68, 0.80)	0.73 (0.67, 0.78)	0.71 (0.66, 0.77)	0.75 (0.69, 0.81)

Cox proportional hazards regression adjusted for age and sex, ethnicity, region, index of multiple deprivation, household income, education, middle degree, drug use, history of cancer screening, family history of diabetes, cancer, or CVD. The variable used for stratification was not adjusted for. Abbreviations: NCD, non-communicable disease; ACS, the American Cancer Society guidelines score; CDRI, chronic disease risk index; ELIH, empirical lifestyle pattern score for hyperinsulinemia; ELIR, empirical lifestyle pattern score for insulin resistance; HB, Health behaviors; HLI, healthy lifestyle index; HLS, healthy lifestyle score; LIS, lifestyle inflammation score; LRLS, low-risk lifestyle score; MEDLIFE, the Mediterranean lifestyle; WCRF/AICR score, World Cancer Research Fund and the American Institute for Cancer Research score.

Supplementary Table 9. Associations of 13 lifestyle scores with major NCD mortality in subgroups

**Subgroup**		**Age**		**Sex**		**Deprivation index**	
**Lifestyle score**	**Category**	**<60**	**≥60**	**Women**	**Men**	**<Median**	**≥Median**
ACS	C1	1.00	1.00	1.00	1.00	1.00	1.00
	C2	0.97 (0.81, 1.16)	0.97 (0.85, 1.11)	1.02 (0.84, 1.23)	0.95 (0.83, 1.08)	1.03 (0.88, 1.21)	0.93 (0.80, 1.08)
	C3	0.87 (0.72, 1.05)	0.89 (0.77, 1.02)	0.89 (0.74, 1.07)	0.89 (0.78, 1.03)	0.91 (0.77, 1.07)	0.87 (0.75, 1.02)
	C4	0.69 (0.54, 0.88)	0.74 (0.63, 0.88)	0.85 (0.70, 1.04)	0.61 (0.50, 0.75)	0.72 (0.59, 0.89)	0.74 (0.61, 0.89)
CDRI	C1	1.00	1.00	1.00	1.00	1.00	1.00
	C2	0.62 (0.51, 0.76)	0.72 (0.63, 0.84)	0.63 (0.53, 0.76)	0.73 (0.63, 0.85)	0.74 (0.61, 0.89)	0.68 (0.58, 0.79)
	C3	0.59 (0.48, 0.72)	0.55 (0.47, 0.64)	0.52 (0.43, 0.63)	0.59 (0.51, 0.69)	0.64 (0.53, 0.76)	0.54 (0.46, 0.63)
	C4	0.50 (0.39, 0.64)	0.52 (0.44, 0.62)	0.55 (0.39, 0.77)	0.52 (0.44, 0.62)	0.56 (0.45, 0.70)	0.49 (0.40, 0.61)
ELIH	C1	1.00	1.00	1.00	1.00	1.00	
	C2	0.92 (0.77, 1.11)	0.80 (0.70, 0.92)	0.89 (0.74, 1.07)	0.82 (0.72, 0.94)	0.83 (0.71, 0.98)	1.00
	C3	0.76 (0.62, 0.93)	0.72 (0.62, 0.83)	0.72 (0.59, 0.87)	0.75 (0.65, 0.87)	0.72 (0.60, 0.85)	0.73 (0.62, 0.85)
	C4	0.73 (0.59, 0.90)	0.68 (0.59, 0.80)	0.76 (0.63, 0.91)	0.64 (0.54, 0.77)	0.70 (0.58, 0.84)	0.70 (0.59, 0.84)
ELIR	C1	1.00	1.00	1.00	1.00	1.00	1.00
	C2	0.78 (0.64, 0.95)	0.84 (0.73, 0.97)	0.81 (0.67, 0.98)	0.83 (0.72, 0.95)	0.82 (0.69, 0.97)	0.82 (0.70, 0.96)
	C3	0.74 (0.60, 0.91)	0.77 (0.66, 0.89)	0.70 (0.58, 0.85)	0.80 (0.69, 0.93)	0.73 (0.61, 0.87)	0.78 (0.66, 0.92)
	C4	0.89 (0.73, 1.08)	0.89 (0.77, 1.02)	0.81 (0.67, 0.97)	0.94 (0.81, 1.10)	0.81 (0.68, 0.97)	0.96 (0.82, 1.13)
HB	C1	1.00	1.00	1.00	1.00	1.00	1.00
	C2	0.70 (0.56, 0.88)	0.71 (0.59, 0.86)	0.71 (0.53, 0.94)	0.71 (0.60, 0.83)	0.81 (0.65, 1.01)	0.64 (0.53, 0.77)
	C3	0.66 (0.53, 0.82)	0.63 (0.52, 0.75)	0.61 (0.46, 0.79)	0.65 (0.55, 0.76)	0.72 (0.58, 0.89)	0.58 (0.48, 0.70)
	C4	0.43 (0.33, 0.56)	0.66 (0.54, 0.80)	0.55 (0.42, 0.73)	0.60 (0.49, 0.73)	0.70 (0.55, 0.89)	0.51 (0.41, 0.62)
HLI	C1	1.00	1.00	1.00	1.00	1.00	1.00
	C2	0.84 (0.70, 1.01)	1.01 (0.88, 1.16)	0.94 (0.78, 1.13)	0.95 (0.83, 1.08)	0.91 (0.78, 1.08)	0.97 (0.84, 1.13)
	C3	0.63 (0.51, 0.76)	0.90 (0.78, 1.03)	0.80 (0.66, 0.96)	0.80 (0.69, 0.92)	0.73 (0.62, 0.87)	0.84 (0.72, 0.99)
	C4	0.63 (0.52, 0.78)	0.78 (0.67, 0.91)	0.81 (0.67, 0.98)	0.66 (0.55, 0.78)	0.79 (0.66, 0.95)	0.69 (0.58, 0.82)
HLI$_{WHR}$	C1	1.00	1.00	1.00	1.00	1.00	1.00
	C2	0.83 (0.68, 1.00)	0.92 (0.80, 1.05)	0.94 (0.75, 1.18)	0.87 (0.77, 0.99)	0.90 (0.76, 1.06)	0.87 (0.75, 1.02)
	C3	0.64 (0.52, 0.78)	0.87 (0.75, 1.01)	0.82 (0.66, 1.02)	0.79 (0.68, 0.91)	0.84 (0.70, 1.00)	0.75 (0.64, 0.89)
	C4	0.59 (0.48, 0.74)	0.82 (0.70, 0.96)	0.81 (0.66, 1.00)	0.66 (0.55, 0.80)	0.83 (0.69, 1.00)	0.67 (0.56, 0.80)
HLI$_{WST}$	C1	1.00	1.00	1.00	1.00	1.00	1.00
	C2	0.75 (0.63, 0.90)	0.90 (0.79, 1.03)	0.79 (0.65, 0.96)	0.87 (0.77, 1.00)	0.86 (0.73, 1.01)	0.84 (0.72, 0.98)
	C3	0.66 (0.54, 0.80)	0.87 (0.75, 1.00)	0.74 (0.61, 0.90)	0.82 (0.71, 0.94)	0.80 (0.68, 0.95)	0.77 (0.65, 0.90)
	C4	0.60 (0.48, 0.75)	0.83 (0.71, 0.97)	0.77 (0.64, 0.94)	0.68 (0.57, 0.82)	0.82 (0.68, 0.99)	0.70 (0.59, 0.84)
HLS	C1	1.00	1.00	1.00	1.00	1.00	1.00
	C2	0.78 (0.66, 0.93)	0.79 (0.69, 0.90)	0.77 (0.64, 0.92)	0.80 (0.70, 0.91)	0.87 (0.74, 1.03)	0.73 (0.64, 0.84)
	C3	0.63 (0.52, 0.77)	0.67 (0.58, 0.77)	0.75 (0.63, 0.90)	0.60 (0.52, 0.69)	0.73 (0.61, 0.86)	0.62 (0.53, 0.73)
	C4	0.46 (0.34, 0.62)	0.57 (0.47, 0.70)	0.62 (0.48, 0.79)	0.48 (0.38, 0.61)	0.64 (0.51, 0.81)	0.46 (0.36, 0.59)

Subgroup		Age		Sex		Deprivation index	
**Lifestyle score**	**Category**	**<60**	**≥60**	**Women**	**Men**	**<Median**	**≥Median**
LIS	C1	1.00	1.00	1.00	1.00	1.00	1.00
	C2	1.04 (0.86, 1.26)	1.06 (0.92, 1.23)	1.01 (0.84, 1.22)	1.09 (0.94, 1.27)	0.99 (0.83, 1.19)	1.13 (0.97, 1.32)
	C3	0.75 (0.62, 0.90)	0.86 (0.75, 0.97)	0.88 (0.74, 1.04)	0.78 (0.69, 0.89)	0.88 (0.75, 1.02)	0.77 (0.66, 0.89)
	C4	0.58 (0.46, 0.72)	0.67 (0.58, 0.79)	0.67 (0.55, 0.81)	0.62 (0.52, 0.74)	0.66 (0.55, 0.80)	0.64 (0.53, 0.76)
LRLS	C1	1.00	1.00	1.00	1.00	1.00	1.00
	C2	0.85 (0.71, 1.02)	0.96 (0.84, 1.09)	0.91 (0.78, 1.07)	0.94 (0.81, 1.08)	1.02 (0.86, 1.20)	0.88 (0.76, 1.01)
	C3	0.71 (0.58, 0.87)	0.82 (0.72, 0.95)	0.72 (0.60, 0.86)	0.83 (0.71, 0.96)	0.87 (0.74, 1.04)	0.72 (0.61, 0.84)
	C4	0.70 (0.54, 0.90)	0.64 (0.52, 0.78)	0.76 (0.55, 1.05)	0.65 (0.54, 0.79)	0.74 (0.59, 0.92)	0.63 (0.50, 0.79)
MEDLIFE	C1	1.00	1.00	1.00	1.00	1.00	1.00
	C2	0.95 (0.78, 1.15)	0.90 (0.78, 1.04)	1.01 (0.83, 1.24)	0.88 (0.76, 1.02)	0.92 (0.77, 1.09)	0.94 (0.80, 1.10)
	C3	0.74 (0.60, 0.91)	0.89 (0.77, 1.03)	0.94 (0.77, 1.15)	0.79 (0.68, 0.92)	0.80 (0.67, 0.95)	0.90 (0.77, 1.07)
	C4	0.75 (0.62, 0.90)	0.82 (0.72, 0.94)	0.87 (0.72, 1.04)	0.77 (0.67, 0.89)	0.81 (0.69, 0.95)	0.80 (0.68, 0.93)
WCRF/AICR	C1	1.00	1.00	1.00	1.00	1.00	1.00
	C2	0.86 (0.71, 1.03)	0.97 (0.83, 1.12)	0.96 (0.78, 1.18)	0.91 (0.79, 1.04)	0.91 (0.77, 1.08)	0.91 (0.77, 1.07)
	C3	0.71 (0.59, 0.85)	0.83 (0.72, 0.96)	0.84 (0.69, 1.03)	0.75 (0.65, 0.86)	0.68 (0.58, 0.80)	0.86 (0.73, 1.00)
	C4	0.59 (0.47, 0.74)	0.82 (0.70, 0.96)	0.78 (0.64, 0.96)	0.71 (0.60, 0.84)	0.65 (0.53, 0.78)	0.83 (0.70, 0.99)

Cox proportional hazards regression adjusted for age and sex, ethnicity, region, index of multiple deprivation, household income, education, middle degree, drug use, history of cancer screening, family history of diabetes, cancer, or CVD. The variable used for stratification was not adjusted for. Abbreviations: NCD, non-communicable disease; ACS, the American Cancer Society guidelines score; CDRI, chronic disease risk index; ELIH, empirical lifestyle pattern score for hyperinsulinemia; ELIR, empirical lifestyle pattern score for insulin resistance; HB, Health behaviors; HLI, healthy lifestyle index; HLS, healthy lifestyle score; LIS, lifestyle inflammation score; LRLS, low-risk lifestyle score; MEDLIFE, the Mediterranean lifestyle; WCRF/AICR score, World Cancer Research Fund and the American Institute for Cancer Research score.

Supplementary References

1. McCullough ML, Patel AV, Kushi LH, et al. Following cancer prevention guidelines reduces risk of cancer, cardiovascular disease, and all-cause mortality. *Cancer epidemiology, biomarkers & prevention : a publication of the American Association for Cancer Research, cosponsored by the American Society of Preventive Oncology* 2011;20(6):1089-97. doi: 10.1158/1055-9965.Epi-10-1173
2. Meng L, Maskarinec G, Lee J, et al. Lifestyle factors and chronic diseases: application of a composite risk index. *Prev Med* 1999;29(4):296-304. doi: 10.1006/pmed.1999.0538
3. Tabung FK, Wang W, Fung TT, et al. Development and validation of empirical indices to assess the insulinaemic potential of diet and lifestyle. *Br J Nutr* 2016;116(10):1787-98. doi: 10.1017/s0007114516003755
4. Khaw KT, Wareham N, Bingham S, et al. Combined impact of health behaviours and mortality in men and women: the EPIC-Norfolk prospective population study. *PLoS Med* 2008;5(1):e12. doi: 10.1371/journal.pmed.0050012
5. Naudin S, Viallon V, Hashim D, et al. Healthy lifestyle and the risk of pancreatic cancer in the EPIC study. *Eur J Epidemiol* 2020;35(10):975-86. doi: 10.1007/s10654-019-00559-6
6. Peila R, Coday M, Crane TE, et al. Healthy lifestyle index and risk of pancreatic cancer in the Women's Health Initiative. *Cancer Causes Control* 2022;33(5):737-47. doi: 10.1007/s10552-022-01558-x
7. Carr PR, Weigl K, Jansen L, et al. Healthy Lifestyle Factors Associated With Lower Risk of Colorectal Cancer Irrespective of Genetic Risk. *Gastroenterology* 2018;155(6):1805-15.e5. doi: 10.1053/j.gastro.2018.08.044
8. Li Z, Gao Y, Byrd DA, et al. Novel Dietary and Lifestyle Inflammation Scores Directly Associated with All-Cause, All-Cancer, and All-Cardiovascular Disease Mortality Risks Among Women. *J Nutr* 2021;151(4):930-39. doi: 10.1093/jn/nxaa388
9. Li Y, Pan A, Wang DD, et al. Impact of Healthy Lifestyle Factors on Life Expectancies in the US Population. *Circulation* 2018;138(4):345-55. doi: 10.1161/circulationaha.117.032047
10. Mata-Fernández A, Hershey MS, Pastrana-Delgado JC, et al. A Mediterranean lifestyle reduces the risk of cardiovascular disease in the "Seguimiento Universidad de Navarra" (SUN) cohort. *Nutrition, metabolism, and cardiovascular diseases : NMCD* 2021;31(6):1728-37. doi: 10.1016/j.numecd.2021.02.022
11. Shams-White MM, Brockton NT, Mitrou P, et al. Operationalizing the 2018 World Cancer Research Fund/American Institute for Cancer Research (WCRF/AICR) cancer prevention recommendations: a standardized scoring system. *Nutrients* 2019;11(7):1572.
12. Piernas C, Perez-Cornago A, Gao M, et al. Describing a new food group classification system for UK biobank: analysis of food groups and sources of macro- and micronutrients in 208,200 participants. *Eur J Nutr* 2021;60(5):2879-90. doi: 10.1007/s00394-021-02535-x
13. Carter JL, Lewington S, Piernas C, et al. Reproducibility of dietary intakes of macronutrients, specific food groups, and dietary patterns in 211 050 adults in the UK Biobank study. *J Nutr Sci* 2019;8:e34. doi: 10.1017/jns.2019.31
